# Assessing Reference Genes for Accurate Transcript Normalization Using Quantitative Real-Time PCR in Pearl Millet [*Pennisetum glaucum* (L.) R. Br.]

**DOI:** 10.1371/journal.pone.0106308

**Published:** 2014-08-29

**Authors:** Prasenjit Saha, Eduardo Blumwald

**Affiliations:** Department of Plant Sciences, University of California Davis, Davis, California, United States of America; National Key Laboratory of Crop Genetic Improvement, China

## Abstract

Pearl millet [*Pennisetum glaucum* (L.) R.Br.], a close relative of Panicoideae food crops and bioenergy grasses, offers an ideal system to perform functional genomics studies related to C4 photosynthesis and abiotic stress tolerance. Quantitative real-time reverse transcription polymerase chain reaction (qRT-PCR) provides a sensitive platform to conduct such gene expression analyses. However, the lack of suitable internal control reference genes for accurate transcript normalization during qRT-PCR analysis in pearl millet is the major limitation. Here, we conducted a comprehensive assessment of 18 reference genes on 234 samples which included an array of different developmental tissues, hormone treatments and abiotic stress conditions from three genotypes to determine appropriate reference genes for accurate normalization of qRT-PCR data. Analyses of Ct values using Stability Index, BestKeeper, ΔCt, Normfinder, geNorm and RefFinder programs ranked *PP2A*, *TIP41*, *UBC2*, *UBQ5* and *ACT* as the most reliable reference genes for accurate transcript normalization under different experimental conditions. Furthermore, we validated the specificity of these genes for precise quantification of relative gene expression and provided evidence that a combination of the best reference genes are required to obtain optimal expression patterns for both endogeneous genes as well as transgenes in pearl millet.

## Introduction

Increasing global population has raised the need of both food and fuel production. In addition, the growing use of fossil fuel is contributing to global climate changes due to elevated greenhouse gas emission. Pearl millet [*Pennisetum glaucum* (L.) R. Br., formerly *P. americanum*] is an excellent food and forage crop of arid to semiarid regions of the world [1], [2] and a close relative of Panicoideae bioenergy grasses like switchgrass and foxtail millet [3]. It is well adapted to drought, heat, high salinity, poor soil fertility and low pH with an efficient C4 carbon fixation and high yield potential [4]. Thereby, pearl millet provides an ideal crop for functional genomics studies related to C4 photosynthesis and abiotic stress tolerance. Although several genetic engineering studies have been conducted in pearl millet [5], [6], functional genomic studies under abiotic stress conditions are scanty [7].

Quantitative real-time polymerase chain reaction (qRT-PCR) provides an important platform for measuring gene expression changes due to its high sensitivity, specificity and wide range of application [8]. However, its accuracy is influenced by the expression stability of the internal control reference genes for reliable transcript normalization of target genes [9], [10]. An ideal reference gene should be constitutively and equally expressed across developmental stages and experimental conditions [9]. According to the ‘golden rules’ [11], identification of the most suitable and highly stable internal reference genes for accurate normalization is one of the prerequisites for qRT-PCR. So far most of the studies published deal with model plant species with known genome sequence, for e.g. Arabidopsis [12], rice [13], brachypodium [14]; however, relatively few studies have been documented in plants with limited or no genome information [15], [16]. Thus the lack of suitable reference genes is one of the major limitations for gene expression studies using qRT-PCR in crop plants [16], including pearl millet.

Over the past few years emphasis has been given to identify and validate suitable reference genes from important plant species such as bamboo [17], barley [18], brachypodium [14], cotton [19], foxtail millet [20], mustard [21], peanut [22], wheat [23], [24] and switchgrass [25]. The commonly used traditional housekeeping reference genes include *actin* (*ACT*), *elongation factor 1α* (*EF1α*), *glyceraldehyde-3-phosphate dehydrogenase* (*GAPDH*), *tubulin* (*TUB*), *ubiquitin-conjugating enzyme (UBC)* and *18S ribosomal RNA* (*18S rRNA*) which are involved in basic cellular processes [26]. Moreover, no single traditional reference gene with stable constant expression across tissues and experimental conditions was found, thus leading to explore additional new reference genes for reliable normalization of qRT-PCR data [26]. Recent reports illustrated that *F-box/kelch-repeat protein* (*F-box*), *phosphoenolpyruvate carboxylase-related kinase* (*PEPKR*), *protein phosphatase 2A* (*PP2A*) and *TIP41-like family protein* (*TIP41*) genes are superior compared to traditional reference genes [17], [21], [22], [27]. Several statistical algorithms, namely, Stability Index [28], ΔCt [29], BestKeeper [30], geNorm [31], NormFinder [32], and RefFinder [33] have been employed for proper validation and stability ranking of the best reference genes for qRT-PCR data normalization in numerous plant species. However, to the best of our knowledge, no systematic analysis for the selection of suitable reference genes for qRT-PCR analysis in pearl millet has been reported. Therefore, a comprehensive validation of reference genes under different experimental conditions for accurate transcript normalization is needed in pearl millet.

In this work, we evaluated 18 potential candidate reference genes in 234 samples from three important pearl millet genotypes using qRT-PCR. Expression patterns of these genes were monitored in tissue samples under different developmental processes, hormone treatments and abiotic stress conditions. Expression stability of these genes was validated using six statistical algorithms in order to assign appropriate reference genes suitable to each experimental condition for accurate transcript normalization. Our results showed that sets of genes are appropriate for accurate transcript quantification of endogenous genes as well as transgenes from different tissue samples. We further illustrated detailed expression patterns of three essential pearl millet endogenous genes specific to development, hormonal stimuli and abiotic stresses.

## Materials and Methods

### Plant materials

Pearl millet (*Pennisetum glaucum* [L.] R. Br.) genotypes ICMR01004, IPCI1466 and IP300088 were used in this study. Seeds of ICMR01004 and IPCI1466 were obtained from the International Crops Research Institute for the Semi-Arid-Tropics (ICRISAT), India, while seeds of IP300088 were acquired from the Germplasm Resources Information Network's (GRIN), USA. Seeds were kept in wide mouth polypropylene bottles (VWR) and stored in a seed vault at 9°C with a relative humidity of 50%.

### Developmental tissue samples

For developmental tissue samples, three genotypes were grown in 5 liter pots containing agronomy mix (equal parts of redwood compost, sand and peat moss) under greenhouse conditions of 16 h day/8 h night photoperiod at 30±2°C until maturity. Plants were watered every alternate day with tap water and fertilized biweekly. Tissue samples of vegetative and reproductive stages included callus 30DPC (days post culture), leaf 7DPS (days post sowing), leaf 15DPS, leaf 30DPS, node, internode, sheath, flag leaf, panicle, peduncle and root of 60DPS plant, and 30DPH (days post harvest) seeds. A total of 108 tissues samples comprising of 12 vegetative and reproductive stages from three genotypes in three biological replicates were harvested by immediate quick freezing in liquid nitrogen in 2 ml SealRite microcentrifuge tubes.

### Hormone treatments

Seeds of 30DPH were soaked in 70% (v/v) ethanol for 1 min followed by washing in 2.5% (v/v) sodium hypochlorite solution containing 0.1% (v/v) Tween 20 for 15 min and rinsed thoroughly with sterile distilled water. Surface sterilized seeds were grown in PhytoCon culture vessels (Phytotechnology Laboratories, Overland Park, KS, USA) containing half strength Murashige and Skoog (MS) medium for 14 days. Seedlings were kept in sucrose free liquid half strength MS medium for 24 h. Seedlings of 15DPG (days post germination) were transferred to PhytoCon culture vessels (Phytotechnology Laboratories) containing liquid half strength MS supplemented with 100 µM abscisic acid (ABA, Sigma, St. Louis, MO, USA), 50 µM brassinolide (Bra, Sigma), 50 µM gibberellic acid (GA, Sigma), 50 µM indole-3-acetic acid (IAA, Sigma), 100 µM methyl jasmonate (MeJa, Sigma), 100 µM salicylic acid (SA, Sigma), 100 µM Zeatin (Zea, Sigma) and incubated for 6 h. Leaves from a total of 72 samples from seven treatments in three biological replicates including one untreated control of three genotypes were harvested and immediately frozen as mentioned in the earlier section.

### Abiotic stress conditions

In the dehydration stress treatments, seedlings of 15DPG (same as hormone treatments) were kept in 400 µM mannitol solution for 6 h. For drought and salinity stresses, water supply was withheld and 300 mM sodium chloride (NaCl) solution was provided for 5 days to 30DPS plants, respectively. Cold and heat stresses were carried out by maintaining 30DPS plants at 4±1°C and 42±1°C, respectively for 6 h for 3 consecutive days. Stress symptoms were monitored visually by the appearance of leaf rolling and yellowing, as well as by measuring stomatal conductance and photosynthesis rates of plants using a LI-COR 6400-40 with an integrated fluorescence chamber head (LI-COR, Lincoln, Nebraska, USA) after the stress treatments.

### Candidate reference genes selection and primer design

Locus identifiers (IDs) of Arabidopsis and rice potential candidate reference genes were obtained from previously published work (Table 1). Orthologous locus IDs from foxtail millet (*Setaria italica*) were identified using locus search from Phytozome. GenBank accession numbers were obtained from National Center for Biotechnology Information (NCBI) using BLASTN.

**Table 1:** Expression level of the selected candidate reference genes tested in pearl millet.

Genes	Description	Arabidopsis	Rice	Foxtail millet/Pearl millet^a^	Ct^b^±SD	CV±SD
*ACT*	Actin	At1g22620	LOC_Os05g36290	Si022372m.g/HM243500	28.2±2.7	1.8±0.5
*CYC*	Cyclophilin, peptidyl-prolyl cis-trans isomerase	At5g35100	LOC_Os08g19610	Si014078m.g	31.5±3.0	3.3±1.0
*eEF1α*	Eukaryotic elongation factor 1 alpha	At5g60390	LOC_Os03g08050	Si022039m.g/EF694165	24.1±3.2	1.8±0.5
*FBX*	F-box domain containing protein	At5g15710	LOC_Os04g57290	Si022138m.g	25.3±2.8	2.6±0.7
*GAPDH*	Glyceraldehyde-3-phosphate dehydrogenase	At3g04120	LOC_Os08g03290	Si014034m.g/GQ398107	22.8±3.1	2.6±0.6
*eIF4a2*	Eukaryotic initiation factor 4a2	At1g54270	N	Si006546m.g/EU856535	23.2±2.1	2.9±0.7
*PEPKR*	Phosphoenolpyruvate carboxylase kinase related	At1g12580	LOC_Os06g03682	Si006273m.g/FR872788	25.6±1.5	1.3±0.4
*PP2A*	Protein phosphatase 2A	At1g10430	LOC_Os02g12580	Si017892m.g	25.6±2.5	1.3±0.3
*RCA*	Rubisco activase	At2g39730	LOC_Os11g47970	Si026414m.g	24.9±2.9	2.4±0.6
*SAMDc*	S-adenosyl methionine decarboxylase	At3g25570	LOC_Os04g42090	Si010282m.g	26.2±5.2	4.8±1.2
*TUA*	Tubulin alpha	At1g04820	LOC_Os03g51600	Si035654m.g	23.1±3.4	2.1±0.5
*TIP41*	Tonoplast intrinsic protein	At4g34270	LOC_Os03g55270	Si036884m.g	28.5±1.5	1.1±0.3
*UBC2*	Ubiquitin-conjugating enzyme 2	At5g25760	LOC_Os02g42314	Si018564m.g	29.8±3.1	1.7±0.5
*UBC18*	Ubiquitin-conjugating enzyme 18	At5g42990	LOC_Os12g44000	Si023498m.g	26.3±2.2	2.7±0.6
*UBQ5*	Ubiquitin 5	At2g47110	LOC_Os01g22490	Si003209m.g	23.5±2.1	1.3±0.4
*UNK*	Transmembrane protein 56	At1g31300	LOC_Os01g56230	Si002525m.g	27.9±1.7	2.6±0.6
*18S rRNA*	18S ribosomal RNA	N	N	KC201690	24.0±4.9	5.7±1.3
*25S rRNA*	25S ribosomal RNA	N	N	AB197128	9.1±1.8	3.6±0.3

a Locus identifiers of selected candidate reference genes for foxtail millet and/or GenBank accession numbers for pearl millet with orthologous from Arabidopsis and rice are listed.

b The expression levels of the candidate genes obtained during qRT-PCR experiments of total samples (n = 234) are presented as mean threshold cycle (Ct) values. SD, standard deviation; CV, coefficient of variance; N, no corresponding locus identifier or accession number.

A total of eighteen genes were chosen for primer design using Primer3Plus software (http://www.bioinformatics.nl/cgi-bin/primer3plus/primer3plus.cgi) [34] considering the parameters specific for qRT-PCR. The sequences with detailed parameters for each primer pair are given in Table S1.

### RNA isolation and cDNA synthesis

A total of 100 mg of frozen plant material was ground to fine powder in a 2 ml SealRite microcentrifuge tube using 3.2 mm stainless steel beads and an automated shaker SO-10M (Fluid Management, Wheeling, IL, USA). Total RNA was isolated from plant samples using the RNeasy plant mini kit (Qiagen, Valencia, CA, USA) according to the manufacturer's procedure. A first set of on-column DNAse I (Qiagen) digestion was carried out during the RNA extraction steps. The integrity of RNA samples were checked by 1% (w/v) agarose gel. The quantity and quality of RNA samples were also checked using a NanoDrop ND-1000 (NanoDrop Technologies, Wilmington, DE, USA). RNA samples with 260/280 ratio between 1.9 to 2.2 and 260/230 ratio between 2.0–2.5 were used for cDNA synthesis. To completely eliminate DNA contamination, 1 µg of total RNA was subjected to gDNA wipeout reaction using the QuantiTect reverse transcription kit (Qiagen) followed by first strand cDNA synthesis in a 20 µl reaction mixture using an optimized blend of oligo-dT and random primers according to manufacturer's instructions and stored at −20°C.

### PCR and qRT-PCR

Specific amplification from cDNA was checked by PCR following the protocol described earlier [35] using 1 µl of cDNA, 10 mM dNTPs, 1 µM each of forward and reverse primers and one unit *Taq* polymerase in a 10 µl total reaction mixture. The amplification program was as follows: 5 min at 95°C; followed by 30 cycles of 95°C for 30 sec, 58°C for 15 sec, 72°C for 30 sec; and a final extension of 72°C for 10 min followed by electrophoresis on 3% (w/v) agarose gel.

For qRT-PCR, cDNAs were diluted to 20 times into a final volume of 400 µl and the reactions were performed as described previously [36] in an optical 96 well plate (Applied Biosystems, Foster City, CA, USA) containing 1 µl of diluted cDNA, 200 nM of each gene specific primer and 2.5 µl of 2X Fast SYBR Green PCR master mix in a 5 µl total volume using a StepOnePlus™ real time PCR system (Applied Biosystems) equipment. The qRT-PCR reactions were conducted following the fast thermal cycles: 50°C for 2 min, 95°C for 20 sec, followed by 40 cycles of 95°C for 3 sec and 60°C for 30 sec. After 40 cycles, the specificity of the amplifications was tested by heating from 60°C to 95°C with a ramp speed of 1.9°C/min, resulting in melting curves. The threshold cycle (Ct) value was automatically determined for each reaction by the real time PCR system with default parameters. Raw data (not baseline corrected) of fluorescence levels and the specificity of the amplicons were checked by qRT-PCR dissociation curve analysis using StepOne Software (v2.3). The baseline correction and linear regression analysis on each amplification curve including the efficiencies (*E*) of the polymerase chain reactions were calculated based on the slope of the line (*E* = 10^slope^), considering an ideal value range (1.8≤ *E*≥2) and correlation (*R^2^*≥0.9) using the LinRegPCR software [37].The final Ct values were the mean of three biological replicates and the coefficient of variance (CV) was calculated to evaluate the variation of Ct values for each gene. Each qRT-PCR reaction set included water as a negative no-template control (NTC) instead of cDNA.

### Analysis for expression stability of reference genes

Five different types of computer-based programs, Stability Index [28], delta (Δ)Ct [29], BestKeeper [30], geNorm [31] and NormFinder [32] methods were used to rank and compare the stability of candidate reference genes across all the experimental sets. For Stability Index, ΔCt, BestKeeper programs, the Ct value for each candidate reference gene was used to determine its relative expression stability. For NormFinder and geNorm, relative expression values were calculated from 2^−ΔΔCt^ using the formula applied before [31]. Overall recommended comprehensive geomean ranking values of the best reference genes were obtained using the ranking results of four algorithms, except Stability Index, in RefFinder [33]. The pairwise variation (Vn/Vn+1) between two sequential normalization factors (NFn and NFn+1) were estimated using geNorm software provided in qBasePlus (v2.4) [38] package for best and minimal number of reference genes needed to calculate an optimal normalization.

### Validation of reference genes

Six genes were chosen to determine their differential expression after accurate normalization across five experimental sets using single and/or best combinations of reference genes (Table S2). Primer design and qRT-PCR reactions were followed as mentioned before. The average Ct value was calculated from three biological replicates and used for relative expression analyses. Normalization of the gene of interest in developmental tissue samples was calculated using the ΔCt values as previously described [12], while relative expression of genes of interest in hormone treatments and abiotic stress conditions was measured as suggested before [39]. The expression fold change value was represented as relative expression (2^−ΔΔCt^). Statistical significant differences in gene expression patterns were evaluated using Tukey's range test in JMP (v7.0.2).

### Transformation of pearl millet

Particle bombardment-mediated transformation of immature zygotic embryo derived calli was carried out using PDS-1000 He biolistic device (Bio-Rad, Hercules, CA) following the protocol described earlier [6]. Zygotic embryos were isolated from surface sterilized seeds and cultured on MS medium supplemented with 2,4-D (2.5 mg/l), maltose (30 g/l), pH 5.8 for callus formation. Particle bombardments were conducted using pCAMBIA1201 and pCAMBIA1302 vectors plasmid DNA (250 ng/shot) precipitated onto 0.6 µm gold particles (Bio-Rad) at a helium pressure of 1,100 psi following the protocol describe previously [6]. Expression of *β-glucuronidase* (*gus*) reporter gene was performed as mentioned earlier [40], while *green fluorescent protein* (*gfp*) expression was monitored using a fluorescence stereomicroscope (Leica MZ FLIII) coupled with a SPOT Insight CCD camera.

## Results

### Identification of candidate reference genes

We found locus identifiers and/or GenBank accession numbers of selected Arabidopsis and rice candidate reference genes from previous published work (Table 1). We identified orthologous locus IDs and/or GenBank accession numbers of these potential candidate reference genes from foxtail millet, a close relative of pearl millet, using orthologous group search in Phytozome and/or BLASTN search in NCBI GenBank. We selected a total of 18 genes for accurate transcripts normalization during gene expression study using qRT-PCR in pearl millet. These genes included both traditional housekeeping as well as several new reference genes namely, *actin (ACT)*, *cyclophilin (CYC)*, *eukaryotic elongation factor 1 alpha (eEF1α)*, *F-box domain containing protein (FBX), glyceraldehyde-3-phosphate dehydrogenase (GAPDH), eukaryotic initiation factor 4a2 (eIF4a2), phosphoenolpyruvate carboxylase-related kinase (PEPKR), protein phosphatase 2A (PP2A)*, *rubisco activase (RCA)*, *S-adenosyl methionine decarboxylase (SAMDc)*, *alpha tubulin (TUA)*, *tonoplast intrinsic protein (TIP41)*, *ubiquitin-conjugating enzyme 2* (*UBC2*), *ubiquitin-conjugating enzyme 18* (*UBC18*), *ubiquitin 5* (*UBQ5*), *transmembrane protein 56* (*UNK*), *18S ribosomal RNA (18S rRNA)* and *25S ribosomal RNA (25S rRNA)* (Table 1).

Due to insufficient availability of sequence information in the NCBI GenBank, in addition to the sequences of pearl millet obtained from Genbank, we used full length transcript sequences from the foxtail millet to design the gene specific primers for qRT-PCR. Primer pairs were designed to anneal near the 3′ end or at the 3′ UTR of each gene using the Primer3Plus software following the parameters: length: 20±3 mer; product size range: 50–200 base pair; melting temperature: 60°C±3°, guanine-cytosine (GC) content: ∼50% including absence for hairpin structures, self-dimers and weak or no self-complementarities at the 3′ end (Table S1).

### Sample size, RNA quality and qRT-PCR conditions

We tested the expression of these potential candidate reference genes and quantified the Ct values using qRT-PCR in a total experimental set of 234 samples (Table 1). These included developmental tissues, hormone treatments and abiotic stress conditions from three pearl millet genotypes ICMR01004, IPCI1466 and IP300088 (Table 2). The developmental tissues experimental set included 108 samples from 12 vegetative and reproductive stages [callus 30DPC, seed 30DPH, leaf 7DPS, leaf 15DPS, leaf 30DPS, and node, internode, sheath, flag leaf, panicle, peduncle and root from 60DPS plants], whereas hormone treatments and abiotic stress conditions included 72 and 54 samples from 8 [control (without treatment), ABA, Bra, GA, IAA, MeJa, SA and Zea] and 6 [control (without stress), dehydration (mannitol), drought (no water), cold, heat and salinity] sets of samples (Tables 2, S3 to S), respectively. The fifth experimental set comprised of Ct values from 78 tissue samples from each of the three pearl millet genotypes (Table 2). We isolated high quantity [368.7±63.3 ng/µl (mean±standard deviation, SD where n = 234)] and quality (average 260/280 ratio of 2.0±0.1 and 260/230 ratio of 2.1±1.6) of total RNA using the guanidinium thiocyanate-based RNeasy plant mini kit. The complete absence of DNA contamination was confirmed by qRT-PCR after two steps of DNAse treatments (first on-column and second gDNA wipeout reaction) for each sample. The reverse transcriptase reactions were primed using an optimized blend of oligo-dT and random primers provided in the kit in order to amplify transcripts from both highly and weakly expressed genes.

**Table 2:** Expression levels of candidate reference genes across four experimental sets of pearl millet.

Genes	Developmental tissues Ct±SD*	Hormone treatments Ct±SD	Abiotic stresses Ct±SD	Genotypes Ct±SD
*ACT*	28.1±2.8	27.6±1.7	29.3±3.5	28.3±0.7
*CYC*	29.8±3.2	33.2±1.1	32.3±3.0	31.7±1.4
*eEF1α*	23.0±3.6	25.3±1.9	24.3±3.1	24.2±0.9
*FBX*	25.1±3.7	24.5±1.3	26.8±2.2	25.4±0.9
*GAPDH*	23.1±3.4	21.6±1.2	24.0±3.6	22.9±1.0
*eIF4a2*	23.0±2.1	22.5±1.1	24.7±2.4	23.3±1.0
*PEPKR*	25.3±1.3	25.4±0.7	26.3±2.3	25.6±0.5
*PP2A*	25.8±2.9	24.8±1.2	26.5±2.6	25.7±0.7
*RCA*	25.8±3.5	24.1±0.9	24.5±3.0	24.8±0.7
*SAMDc*	27.0±5.5	24.9±4.0	26.6±5.8	26.2±0.9
*TUA*	22.8±3.9	21.4±1.0	26.0±2.8	23.4±1.9
*TIP41*	28.3±1.7	28.1±0.7	29.3±1.7	28.6±0.5
*UBC2*	29.9±3.3	28.8±2.0	30.8±3.5	29.8±0.8
*UBC18*	26.3±2.7	25.8±0.7	26.9±2.4	26.3±0.4
*UBQ5*	23.7±2.5	22.3±0.8	24.7±2.0	23.5±1.0
*UNK*	27.7±2.0	27.6±1.1	28.7±1.7	28.0±0.5
*18S rRNA*	22.5±5.4	26.4±3.0	23.4±5.0	24.1±1.7
*25S rRNA*	9.0±2.3	8.7±0.6	9.8±1.9	9.1±0.5

*, Data are represented as mean threshold cycle (Ct) values from all analyzed samples in each individual experimental set with standard deviation (SD).

### Accuracy and efficiency of amplification

To determine the accuracy of primers designed in this study to specifically amplify potential target candidate reference genes we performed PCR and qRT-PCR using either crude and/or diluted cDNAs. We obtained a single amplified product of the expected size in agarose gel electrophoresis (Figure S1) and the presence of one dominant peak of the specific amplicon in melt curve analysis (Figure S2), respectively. Further, a two-step qRT-PCR protocol for cDNA synthesis and cDNA amplification in successive steps reduced the undesired primer dimer formation using SYBR Green. No detectable amplifications in the no-template controls (NTCs) confirmed the absence of primer dimers or non-specific products (Figure S2). We determined the PCR efficiency (*E*) of each primer pair from the amplification plots of all amplification profiles using LinRegPCR software. The mean *E* values with SD for all primer pairs across all experimental samples from three biological replicates are given in Table S1. Primer pairs of most of the genes exhibited no significant differences in E values and displayed PCR efficiencies of more than 1.90, while primer pair for *CYC* and *25S rRNA* showed PCR efficiencies of 1.87±0.03 and 1.85±0.04 (Table S1). We further calculated correlation coefficients (*R^2^*) of PCR efficiency values to evaluate the amplification curves. Except *TAU* (*R^2^* = 0.89), the rest of the primer pairs revealed *R^2^*>0.90 from all reactions (Table S1).

### Expression levels of candidate reference genes

Expression levels of all the candidate reference genes were measured by monitoring the Ct values in the qRT-PCR reactions. We analyzed all the Ct values under five groups which included total [first experimental set (n = 234), Table 1], developmental tissues [second experimental set (n = 108), Tables 2 and S3], hormone treatments [third experimental set (n = 72), Tables 2 and S4], abiotic stress conditions [fourth experimental set (n = 54), Tables 2 and S5] and genotypes [fifth experimental set (n = 78), Table 2]. In the first total experimental set the mean Ct values of the 18 candidate reference genes revealed a minimum of 9.1±1.8 and a maximum of 31.5±3.0 for highest and lowest expression levels for *25S rRNA* and *CYC* genes, respectively, while most of the values were distributed between 22.8±3.1 to 29.8±3.1 (Table 1). The mean Ct values of *SAMDc* (26.2±5.2) with highest SD indicated less stability as compared to *TIP41* (28.5±1.5) with lowest SD showing relatively stable expression in the total experimental set (Table 1). Similarly, we noticed large SDs of Ct values for *SAMDc* and *18S rRNA* indicating a more variable expression in the other four experimental sets, while little variation of Ct values was detected for rest of the genes (Table 2). In the second experimental set, high Ct values suggested low expression of all the candidate reference genes in the seeds compared to other developmental tissues (Table S3). The third and fourth experimental sets revealed elevated Ct values of these genes under SA treatment (Table S4), and heat and salinity stress conditions (Table S5) as compared to their respective controls. Furthermore, we evaluated the expression levels of candidate reference genes by calculating the CV of the Ct values. Among the four experimental sets, *TIP41* showed the lowest CV value (1.1±0.3), while *SAMDc* and *18S rRNA* revealed a greater variation in expression levels due to their high CV values (4.8±1.2 and 5.7±1.3, Table 1).

### Stability ranking of the candidate reference genes

We used Stability Index (SI), BestKeeper, ΔCt, NormFinder, geNorm and RefFinder programs to identify the best reference genes for qRT-PCR data normalization in pearl millet. These programs allowed us to establish a stability ranking of each candidate reference gene using Ct values across the experimental sets and condition-specific levels (Tables 3–7).

**Table 3:** Stability ranking of the reference genes in all tissue and experimental sets studied in pearl millet.

Rank	Stability Index		BestKeeper		ΔCT		NormFinder		geNorm		RefFinder	
	Genes	Index Value (SI)	Genes	CP(%)±SD	Genes	Ave of STDEV	Genes	Stability Value (SV)	Genes	Normalization Value (MV)	Genes	Geomean of ranking values
1	*PEPKR*	0.05	*PEPKR*	2.72±0.69	*PP2A*	1.32	*PP2A*	0.43	*PP2A | TIP41*	0.46	*PP2A*	1.68
2	*PP2A*	0.06	*TIP41*	3.16±0.90	*TIP41*	1.39	*TIP41*	0.57			*TIP41*	2.63
3	*TIP41*	0.09	*PP2A*	3.24±0.90	*UBC2*	1.40	*PEPKR*	0.61	*UBC2*	0.49	*UBC2*	3.41
4	*UBC2*	0.21	*UBC2*	4.24±1.26	*TUA*	1.41	*UBC2*	0.61	*PEPKR*	0.73	*PEPKR*	4.12
5	*TUA*	0.21	*ACT*	4.26±1.20	*eEF1α*	1.42	*UBC18*	0.68	*UBQ5*	0.83	*UBQ5*	4.16
6	*eIF4a2*	0.23	*TUA*	4.48±1.17	*UBQ5*	1.48	*eEF1α*	0.82	*eEF1α*	0.88	*eEF1α*	5.63
7	*ACT*	0.29	*eIF4a2*	4.60±1.07	*ACT*	1.51	*UBQ5*	0.86	*ACT*	0.93	*UBC18*	6.40
8	*UBQ5*	0.31	*eEF1α*	4.90±1.25	*eIF4a2*	1.55	*eIF4a2*	0.93	*UBC18*	0.96	*TUA*	6.45
9	*UBC18*	0.33	*UNK*	5.57±1.46	*PEPKR*	1.56	*ACT*	0.94	*TUA*	0.99	*ACT*	7.33
10	*eEF1α*	0.39	*UBQ5*	5.57±1.31	*GAPDH*	1.69	*TUA*	1.16	*GAPDH*	1.04	*eIF4a2*	7.94
11	*SAMDc*	0.67	*GAPDH*	6.76±1.54	*SAMDc*	1.72	*SAMDc*	1.17	*eIF4a2*	1.09	*GAPDH*	10.47
12	*GAPDH*	0.68	*UBC18*	7.14±1.81	*UNK*	1.73	*UNK*	1.18	*UNK*	1.15	*UNK*	11.00
13	*UNK*	0.83	*RCA*	7.26±1.80	*FBX*	1.87	*FBX*	1.40	*FBX*	1.22	*FBX*	13.47
14	*FBX*	0.86	*SAMDc*	7.46±1.79	*25S rRNA*	1.96	*25S rRNA*	1.45	*25S rRNA*	1.31	*RCA*	13.74
15	*RCA*	0.87	*CYC*	7.51±2.36	*UBC18*	2.22	*GAPDH*	1.86	*SAMDc*	1.40	*SAMDc*	15.24
16	*25S rRNA*	1.02	*FBX*	10.18±2.35	*RCA*	2.53	*RCA*	2.22	*RCA*	1.53	*25S rRNA*	15.47
17	*CYC*	1.83	*25S rRNA*	10.24±0.93	*CYC*	2.72	*CYC*	2.42	*CYC*	1.67	*CYC*	17.00
18	*18S rRNA*	1.97	*18S rRNA*	10.95±2.62	*18S rRNA*	2.84	*18S rRNA*	2.59	*18S rRNA*	1.80	*18S rRNA*	18.00

CP, crossing point; STDEV and SD, standard deviation.

**Table 4:** Stability ranking of the reference genes in developmental tissues of pearl millet.

Rank	Stability Index		BestKeeper		ΔCT		Normfinder		geNorm		RefFinder	
	Genes	Index value (SI)	Genes	CP(%)±SD	Genes	Ave of STDEV	Genes	Stability Value (SV)	Genes	Normalization Value (MV)	Genes	Geomean of ranking values
1	*PEPKR*	0.06	*PEPKR*	2.78±0.70	*UBC2*	1.33	*TIP41*	0.31	*PP2A | TIP41*	0.32	*PP2A*	2.28
2	*TIP41*	0.16	*UBC2*	4.02±1.14	*PP2A*	1.34	*PP2A*	0.38			*TIP41*	2.51
3	*PP2A*	0.17	*eEF1α*	4.27±1.18	*TUA*	1.41	*UBC2*	0.52	*UBC2*	0.36	*UBC2*	3.72
4	*eEF1α*	0.23	*PP2A*	4.66±1.07	*UBQ5*	1.43	*PEPKR*	0.66	*PEPKR*	0.46	*TUA*	4.46
5	*ACT*	0.35	*ACT*	5.02±1.41	*eEF1α*	1.44	*eEF1α*	0.69	*eEF1α*	0.64	*eEF1α*	5.42
6	*UBC2*	0.36	*TIP41*	5.10±1.52	*TIP41*	1.48	*TUA*	0.76	*ACT*	0.79	*ACT*	5.44
7	*TUA*	0.57	*UBQ5*	6.21±1.43	*UBC18*	1.48	*ACT*	0.81	*UBQ5*	0.86	*PEPKR*	6.45
8	*UNK*	0.63	*eIF4a2*	6.26±1.61	*PEPKR*	1.50	*UBQ5*	0.81	*TUA*	0.94	*UBQ5*	6.97
9	*UBQ5*	0.63	*TUA*	6.29±1.69	*ACT*	1.53	*eIF4a2*	0.82	*UNK*	0.98	*UBC18*	7.48
10	*eIF4a2*	0.68	*UNK*	6.32±1.49	*GAPDH*	1.68	*UNK*	1.11	*GAPDH*	1.02	*UNK*	7.64
11	*UBC18*	0.73	*UBC18*	6.60±1.73	*25S rRNA*	1.70	*GAPDH*	1.12	*25S rRNA*	1.09	*eIF4a2*	7.67
12	*CYC*	1.04	*GAPDH*	7.70±1.77	*eIF4a2*	1.76	*25S rRNA*	1.27	*eIF4a2*	1.15	*25S rRNA*	8.82
13	*GAPDH*	1.08	*CYC*	8.03±2.39	*18S rRNA*	1.95	*UBC18*	1.55	*UBC18*	1.22	*GAPDH*	10.94
14	*18S rRNA*	1.70	*18S rRNA*	8.52±1.91	*UNK*	1.99	*18S rRNA*	1.59	*18S rRNA*	1.30	*18S rRNA*	13.74
15	*FBX*	1.83	*FBX*	9.62±2.42	*SAMDc*	2.23	*SAMDc*	1.88	*FBX*	1.40	*SAMDc*	15.24
16	*25S rRNA*	2.09	*SAMDc*	10.45±2.38	*FBX*	2.24	*FBX*	1.94	*SAMDc*	1.48	*FBX*	15.98
17	*RCA*	2.31	*RCA*	11.56±2.94	*CYC*	2.75	*CYC*	2.46	*CYC*	1.63	*CYC*	16.74
18	*SAMDc*	2.40	*25S rRNA*	13.08±1.17	*RCA*	3.22	*RCA*	3.02	*RCA*	1.80	*RCA*	18.00

CP, crossing point; STDEV and SD, standard deviation.

**Table 5:** Stability ranking of the reference genes in hormone treatments of pearl millet.

Rank	Stability Index		BestKeeper		ΔCT		Normfinder		geNorm		RefFinder	
	Genes	Index value (SI)	Genes	CP(%)±SD	Genes	Ave of STDEV	Genes	Stability Value (SV)	Genes	Normalization Value (MV)	Genes	Geomean of ranking values
1	*TIP41*	0.02	*TUA*	0.70±0.23	*TIP41*	0.56	*TIP41*	0.13	*TIP41 | UBQ5*	0.16	*TIP41*	1.73
2	*ACT*	0.02	*TIP41*	0.88±0.25	*PP2A*	0.57	*PEPKR*	0.16			*UBQ5*	2.91
3	*PEPKR*	0.02	*UBQ5*	0.91±0.26	*UBC2*	0.57	*UBQ5*	0.18	*PP2A*	0.17	*PP2A*	4.43
4	*TUA*	0.05	*ACT*	1.02±0.23	*TUA*	0.59	*PP2A*	0.18	*ACT*	0.17	*PEPKR*	4.61
5	*UBQ5*	0.06	*UBC2*	1.06±0.27	*PEPKR*	0.60	*eEF1α*	0.20	*eEF1α*	0.21	*ACT*	4.68
6	*UBC2*	0.06	*eEF1α*	1.07±0.29	*eEF1α*	0.62	*ACT*	0.22	*UBC2*	0.24	*eEF1α*	5.13
7	*eEF1α*	0.06	*PP2A*	1.14±0.28	*ACT*	0.63	*eIF4a2*	0.26	*PEPKR*	0.26	*TUA*	6.70
8	*PP2A*	0.07	*UBC18*	1.24±0.32	*UBQ5*	0.64	*UBC2*	0.33	*TUA*	0.27	*UBC2*	7.00
9	*UNK*	0.07	*eIF4a2*	1.27±0.31	*eIF4a2*	0.65	*TUA*	0.34	*GAPDH*	0.29	*eIF4a2*	7.43
10	*25S rRNA*	0.10	*GAPDH*	1.37±0.30	*GAPDH*	0.66	*UBC18*	0.41	*UBC18*	0.31	*CYC*	7.65
11	*eIF4a2*	0.14	*PEPKR*	1.65±0.35	*UNK*	0.67	*GAPDH*	0.46	*eIF4a2*	0.32	*UBC18*	7.88
12	*FBX*	0.16	*CYC*	1.76±0.39	*25S rRNA*	0.67	*CYC*	0.46	*25S rRNA*	0.34	*GAPDH*	9.12
13	*GAPDH*	0.20	*SAMDc*	2.02±0.50	*CYC*	0.74	*25S rRNA*	0.48	*CYC*	0.38	*25S rRNA*	11.06
14	*UBC18*	0.20	*RCA*	2.14±0.59	*FBX*	0.97	*SAMDc*	0.70	*FBX*	0.43	*SAMDc*	14.73
15	*RCA*	0.46	*GAPC2*	2.79±0.68	*SAMDc*	0.98	*GAPC2*	0.83	*UNK*	0.49	*FBX*	14.73
16	*CYC*	0.47	*25S rRNA*	3.28±0.28	*UBC18*	1.06	*RCA*	0.95	*SAMDc*	0.55	*UNK*	15.49
17	*18S rRNA*	1.57	*UNK*	3.60±0.91	*RCA*	1.49	*UNK*	1.37	*RCA*	0.66	*RCA*	17.00
18	*SAMDc*	4.93	*18S rRNA*	5.04±1.33	*18S rRNA*	2.07	*18S rRNA*	2.05	*18S rRNA*	0.82	*18S rRNA*	18.00

CP, crossing point; STDEV and SD, standard deviation.

**Table 6:** Stability ranking of the reference genes in abiotic stress conditions of pearl millet.

Rank	Stability Index		BestKeeper		ΔCT		Normfinder		geNorm		RefFinder	
	Genes	Index value (SI)	Genes	CP(%)±SD	Genes	Ave of STDEV	Genes	Stability Value (SV)	Genes	Normalization Value (MV)	Genes	Geomean of ranking values
1	*TIP41*	0.18	*TIP41*	1.60±0.47	*UBQ5*	0.97	*TIP41*	0.28	*PP2A | TIP41*	0.39	*PP2A*	1.50
2	*TUA*	0.25	*eEF1α*	2.42±0.60	*TIP41*	1.01	*PP2A*	0.35			*TIP41*	2.94
3	*UBQ5*	0.50	*TUA*	2.65±0.76	*PP2A*	1.03	*UBQ5*	0.35	*UBQ5*	0.42	*UBQ5*	3.36
4	*eEF1α*	0.52	*UBQ5*	2.76±0.68	*ACT*	1.03	*TUA*	0.47	*ACT*	0.47	*ACT*	4.23
5	*PP2A*	0.56	*PP2A*	3.48±0.85	*eEF1α*	1.10	*ACT*	0.64	*TUA*	0.56	*TUA*	4.36
6	*UBC2*	0.59	*PEPKR*	3.49±0.92	*UBC2*	1.14	*eEF1α*	0.73	*UBC2*	0.63	*eEF1α*	4.41
7	*UBC18*	0.84	*UBC2*	3.75±1.00	*PEPKR*	1.17	*UBC2*	0.74	*eEF1α*	0.69	*UBC2*	5.24
8	*PEPKR*	0.86	*UNK*	3.81±0.89	*TUA*	1.19	*eIF4a2*	0.79	*PEPKR*	0.74	*PEPKR*	6.96
9	*ACT*	1.07	*eIF4a2*	4.01±1.30	*GAPDH*	1.25	*PEPKR*	0.88	*UBC18*	0.81	*UBC18*	8.32
10	*UNK*	1.15	*FBX*	4.17±1.12	*eIF4a2*	1.31	*UNK*	0.94	*18S rRNA*	0.88	*GAPDH*	9.46
11	*RCA*	1.67	*CYC*	4.44±1.36	*UBC18*	1.32	*25S rRNA*	0.97	*25S rRNA*	0.93	*18S rRNA*	12.26
12	*eIF4a2*	1.79	*UBC18*	4.61±1.24	*25S rRNA*	1.39	*18S rRNA*	1.09	*UNK*	0.98	*25S rRNA*	12.74
13	*FBX*	2.00	*ACT*	4.96±1.45	*UNK*	1.42	*RCA*	1.10	*GAPDH*	1.02	*UNK*	12.85
14	*CYC*	2.09	*SAMDc*	5.52±1.47	*RCA*	1.42	*SAMDc*	1.13	*RCA*	1.06	*eIF4a2*	13.45
15	*25S rRNA*	2.17	*18S rRNA*	5.78±1.50	*18S rRNA*	1.57	*GAPDH*	1.33	*eIF4a2*	1.12	*RCA*	13.93
16	*GAPDH*	3.29	*RCA*	5.99±1.47	*SAMDc*	1.63	*UBC18*	1.41	*SAMDc*	1.18	*SAMDc*	14.74
17	*18S rRNA*	10.59	*GAPDH*	8.04±1.93	*FBX*	1.75	*FBX*	1.54	*FBX*	1.24	*CYC*	16.26
18	*SAMDc*	12.24	*25S rRNA*	9.14±0.89	*CYC*	1.83	*CYC*	1.62	*CYC*	1.31	*FBX*	17.24

CP, crossing point; STDEV and SD, standard deviation.

**Table 7:** Stability ranking of the reference genes among three genotypes of pearl millet.

Rank	Stability Index		BestKeeper		ΔCT		Normfinder		geNorm		RefFinder	
	Genes	Index value (SI)	Genes	CP(%)±SD	Genes	Ave of STDEV	Genes	Stability Value (SV)	Genes	Normalization Value (MV)	Genes	Geomean of ranking values
1	*TIP41*	0.16	*ACT*	1.09±0.29	*TIP41*	0.64	*TIP41*	0.03	*TIP41 | ACT*	0.05	*TIP41*	1.50
2	*UBQ5*	0.16	*TIP41*	1.31±0.37	*ACT*	0.65	*PP2A*	0.03			*ACT*	2.38
3	*TUA*	0.24	*UBQ5*	1.33±0.38	*eEF1α*	0.65	*ACT*	0.03	*PP2A*	0.08	*PP2A*	2.91
4	*ACT*	0.42	*PEPKR*	1.34±0.34	*TUA*	0.66	*eEF1α*	0.04	*TUA*	0.11	*TUA*	3.31
5	*PEPKR*	0.59	*UBC2*	1.74±0.52	*PP2A*	0.67	*TUA*	0.05	*PEPKR*	0.15	*eEF1α*	3.98
6	*eEF1α*	0.62	*eEF1α*	1.76±0.50	*UBQ5*	0.67	*PEPKR*	0.06	*eEF1α*	0.18	*PEPKR*	5.18
7	*PP2A*	0.78	*PP2A*	1.79±0.46	*UBC2*	0.69	*UBQ5*	0.27	*UBQ5*	0.22	*UBQ5*	6.19
8	*UBC2*	1.00	*RCA*	2.12±0.53	*PEPKR*	0.71	*UBC2*	0.35	*UBC2*	0.25	*UBC2*	7.74
9	*UBC18*	1.19	*TUA*	2.40±0.63	*FBX*	0.74	*FBX*	0.39	*FBX*	0.29	*FBX*	10.05
10	*FBX*	1.19	*UBC18*	2.55±0.62	*25S rRNA*	0.75	*25S rRNA*	0.39	*UBC18*	0.31	*eIF4a2*	11.07
11	*RCA*	1.26	*FBX*	2.61±0.66	*UBC18*	0.78	*UNK*	0.54	*UNK*	0.33	*UBC18*	11.24
12	*CYC*	1.32	*UNK*	2.72±0.64	*GAPDH*	0.79	*GAPDH*	0.56	*GAPDH*	0.35	*GAPDH*	12.24
13	*25S rRNA*	1.68	*GAPDH*	2.82±0.64	*UNK*	1.04	*UBC18*	0.87	*eIF4a2*	0.41	*UNK*	12.47
14	*UNK*	1.85	*eIF4a2*	2.90±0.68	*RCA*	1.19	*RCA*	1.00	*25S rRNA*	0.48	*RCA*	12.54
15	*eIF4a2*	2.01	*CYC*	3.25±1.03	*eIF4a2*	1.34	*eIF4a2*	1.13	*RCA*	0.60	*25S rRNA*	13.77
16	*GAPDH*	2.42	*25S rRNA*	3.57±0.33	*SAMDc*	1.57	*SAMDc*	1.47	*SAMDc*	0.70	*SAMDc*	16.21
17	*SAMDc*	9.55	*18S rRNA*	4.80±1.16	*CYC*	1.66	*CYC*	1.53	*CYC*	0.82	*CYC*	16.74
18	*18S rRNA*	9.86	*SAMDc*	5.73±1.34	*18S rRNA*	2.10	*18S rRNA*	2.05	*18S rRNA*	0.96	*18S rRNA*	17.74

CP, crossing point; STDEV and SD, standard deviation.

The SI was calculated from the multiplication of the slope (a value of the regression analysis of geomeans and overall means) with CV considering the fact that gene with lowest SI from low slope and low CV provided the best reference gene. In the first total experimental set, *PEPKR, PP2A* and *TIP41* with SI values of 0.05, 0.06 and 0.09 were the top three candidates, respectively, whereas *18S rRNA* with highest SI value of 1.97 was the least preferred choice of reference gene (Table 3). Based on SI values, *PEPKR* (SI of 0.06) and *TIP41* (SI of 0.16) were the two best candidates, while *SAMDc* (SI of 2. 40) was the worst candidate for normalization of gene expression in developmental tissue samples (Table 4). Analysis of SI values of reference genes in the third and fourth experimental sets revealed *TIP41* as the superior candidate with the smallest SI of 0.02 and 0.18, respectively for transcript normalization under hormone treatments and abiotic stress conditions (Tables 5 and 6). Among the three genotypes of pearl millet, *TIP41* (SI of 0.16) was the top ranked reference gene for normalization of gene expression (Table 7).

The BestKeeper program determines the stability ranking of the reference genes based on the percentage of crossing point (%CP) to the BestKeeper Index and the SD from the geometric mean of the candidate reference genes Ct values, where the genes with lowest CP and SD values are identified as the best reference genes for normalization. In this study, BestKeeper analyses of the total experimental samples identified *PEPKR* (2.72±0.69), *TIP41* (3.16±0.90) and *PP2A* (3.24±0.90) with lowest CP±SD values (Table 3), where genes with SD<1 are considered as stable. In the developmental tissues, hormone treated, abiotic stressed and genotype experimental sets many genes showed SD<1, while the most stable reference genes were *PEPKR* (2.78±0.70), *TUA* (0.70±0.23), *TIP41* (1.60±0.47) and *ACT* (1.09±0.29), respectively (Tables 4–7).

The ΔCt method compared the relative expression of a reference gene with other candidate reference genes within each sample, thereby ranked the genes based on the average of STDEV or SD. Analyses using this program exhibited *PP2A*, *UBC2*, *TIP41*, *UBQ5* and *TIP41* with average STDEV values of 1.32, 1.33, 0.56, 0.97 and 0.64 as the most suitable reference genes for normalization in total, developmental, hormone treated, abiotic stress and genotypes experimental sets of pearl millet, respectively (Tables 3–7).

NormFinder ranks all candidate reference genes based on intra- and inter-group variations of expression stabilities by measuring the stability value (SV) for each reference gene. In our study, the NormFinder identified *PP2A*, *TIP41* and *PEPKR* with SV of 0.43, 0.57 and 0.61, as the top three optimal reference genes for transcript normalization in the total tissue samples (Table 3). The NormFinder analyses in the developmental tissues (SV of 0.31), hormone treatments (SV of 0.13), abiotic stress conditions (SV of 0.28) and genotypes (SV of 0.03) experimental sets of pearl millet recognized *TIP41* as the most suitable reference gene (Tables 4–7).

In addition, we also examined the stability ranking of candidate reference genes using geNorm program (Tables 3–7). The geNorm statistical algorithm determines the normalization value (MV) based on the geometric mean of multiple reference genes and mean pair-wise variation of a gene from all other reference genes in each set of samples. In both first and second experimental sets, the two best reference genes were *PP2A*| *TIP41* with the lowest MV of 0.46 and 0.32, whereas *UBC2* with MV of 0.49 and 0.36 remained the third most suitable gene for transcript normalization in total and developmental tissues, respectively, as determined by the geNorm (Tables 3–4). The most preferred genes for normalization in hormone treatments and abiotic stress conditions were *TIP41*|*UBQ5* (MV of 0.16) and *PP2A*|*TIP41* (MV of 0.39), respectively (Tables 5–6), while *TIP41*|*ACT* had the lowest MV of 0.05 in the genotypes of pearl millet (Table 7). In addition, geNorm analyses revealed significantly high stability of several reference genes with MV of less than the cut-off range of 1.5 (Tables 3–7).

We further compared all the data generated by SI, BestKeeper, ΔCt, NormFinder and geNorm programs using recommended comprehensive ranking method in RefFinder software to confirm the stability ranking of reference genes for accurate transcript normalization across the experimental sets (Tables 3–7). The overall ranking of the best reference genes in total and categorized experimental sets according to RefFinder are given in Tables 3–8.

**Table 8:** Summary of the best combination of reference genes for accurate normalization across five experimental sets of pearl millet using geNorm and RefFinder programs.

Experimental sets	Total	Development tissues	Hormone treatments	Abiotic stresses	Genotypes
Best combination	V2/3	V3/4	V2/3	V3/4	V2/3
Pairwise variation (V)^a^	0.070	0.138	0.056	0.090	0.110
Reference control genes	*PP2A*	*PP2A*	*TIP41*	*PP2A*	*TIP41*
	*TIP41*	*TIP41*	*UBQ5*	*TIP41*	*ACT*
		*UBC2*		*UBQ5*	

a Pairwise variation (V) represents the optimal combination of reference control genes required to pass the suggested cut-off value 0.15 [31]. A single common reference control gene for expression study across experimental sets is highlighted in gray.

**Figure 1:**
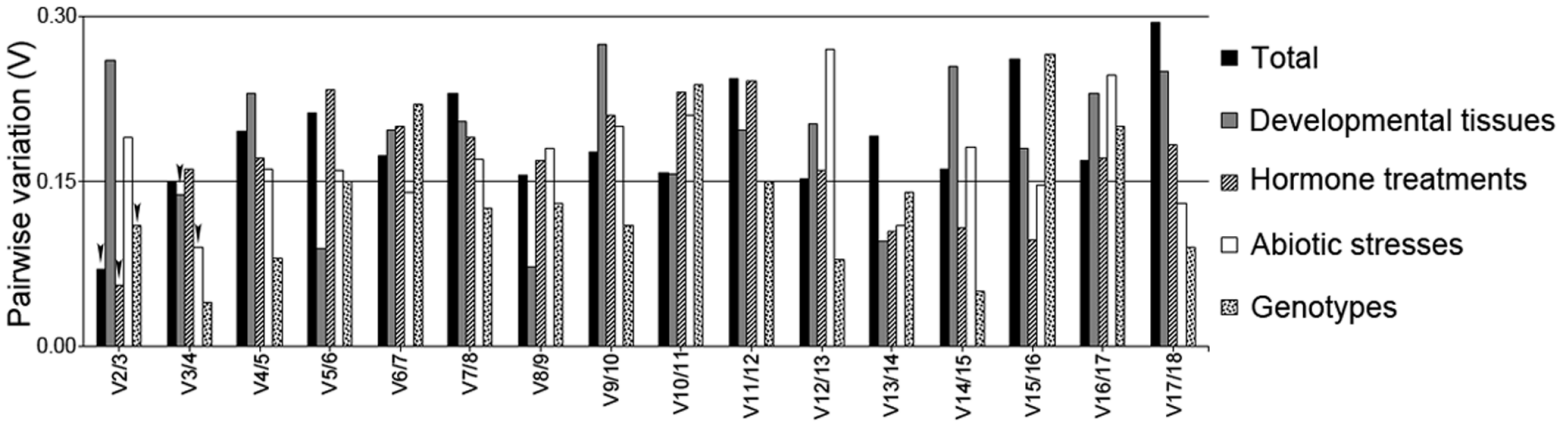
Estimation of pairwise variation to determine the optimal number of control reference genes required for accurate normalization using geNorm. Pairwise variation (V, Vn/Vn+1) was calculated between successively ranked normalization factors NFn and NFn+1. Arrowheads on the bar graph indicate the minimum number of genes required at the cut-off value 0.15 [31]. The V between the normalization factors of the two first-ranked and the three first-ranked is represented by V2/3 and so on, respectively.

We next applied the geNorm software to calculate the Vn/Vn+1 between NFn and NFn+1 to determine the best combination of reference genes required for precise transcript quantification across different sets of experiments. Figure 1 summarizes the V values from the combination of reference genes and shows that a number of genes are required for reliable normalization of gene expression data among different experimental sets (Table 8).

### Accurate normalization of gene and transgene expression using optimal combination of reference genes

**Figure 2:**
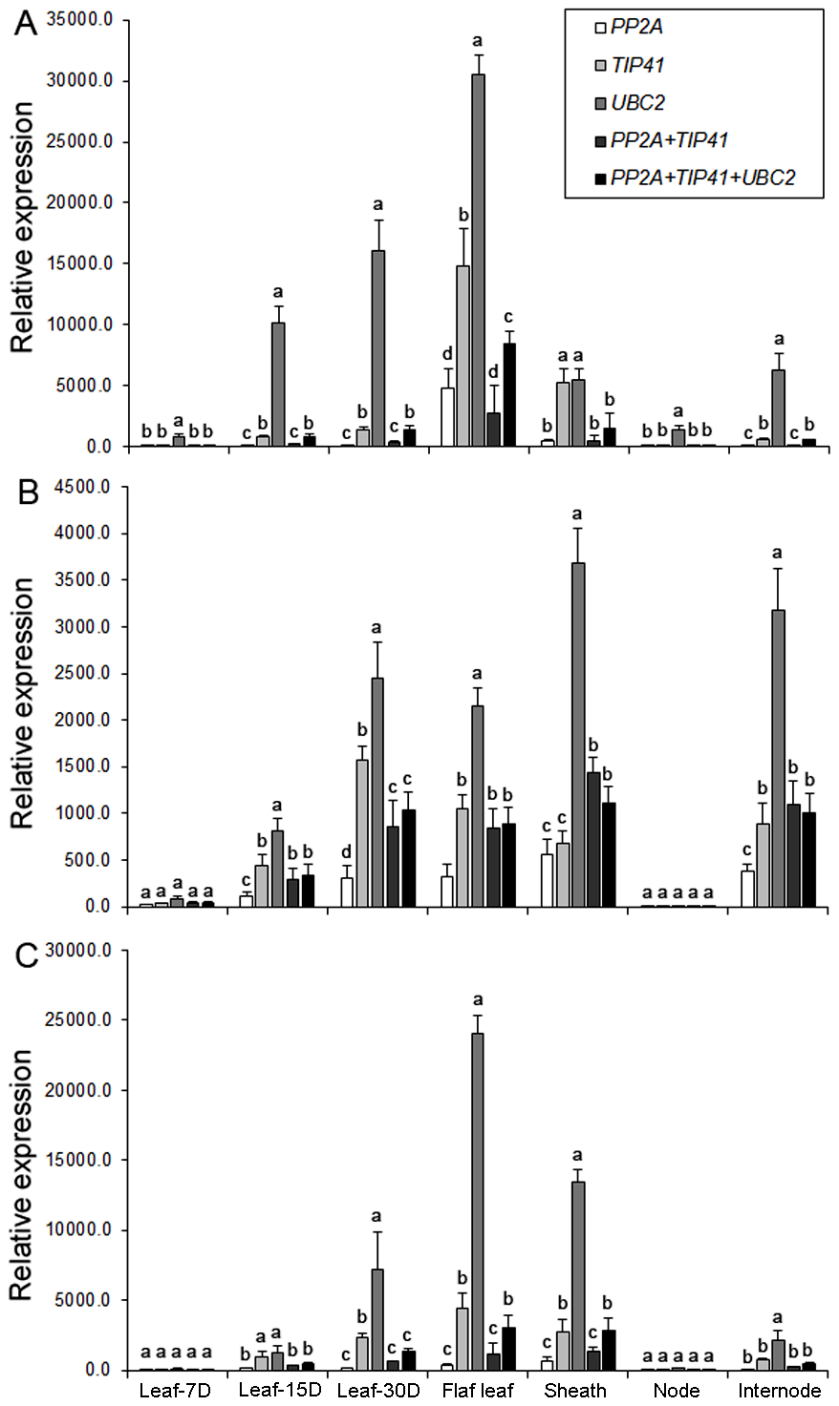
Validation of *PEPC* gene expression after normalization using optimal number of control reference genes in developmental tissue samples from genotypes (A) ICMR01004, (B) IPCI1466 and (C) IP300088. Results are presented as mean relative expression with SD from three biological replicates after normalization using the best combination of reference genes recommended by geNorm and RefFinder (see Table 8) for developmental tissue samples. Leaf- 7D, 15D and 30D represent 7DPS, 15DPS and 30DPS leaf samples while flag leaf, sheath, node and internode are from 60DPS plants. Different letters on the bars indicate significant differences at the *P*≤0.05 level as tested by Tukey's Range Test.

**Figure 3:**
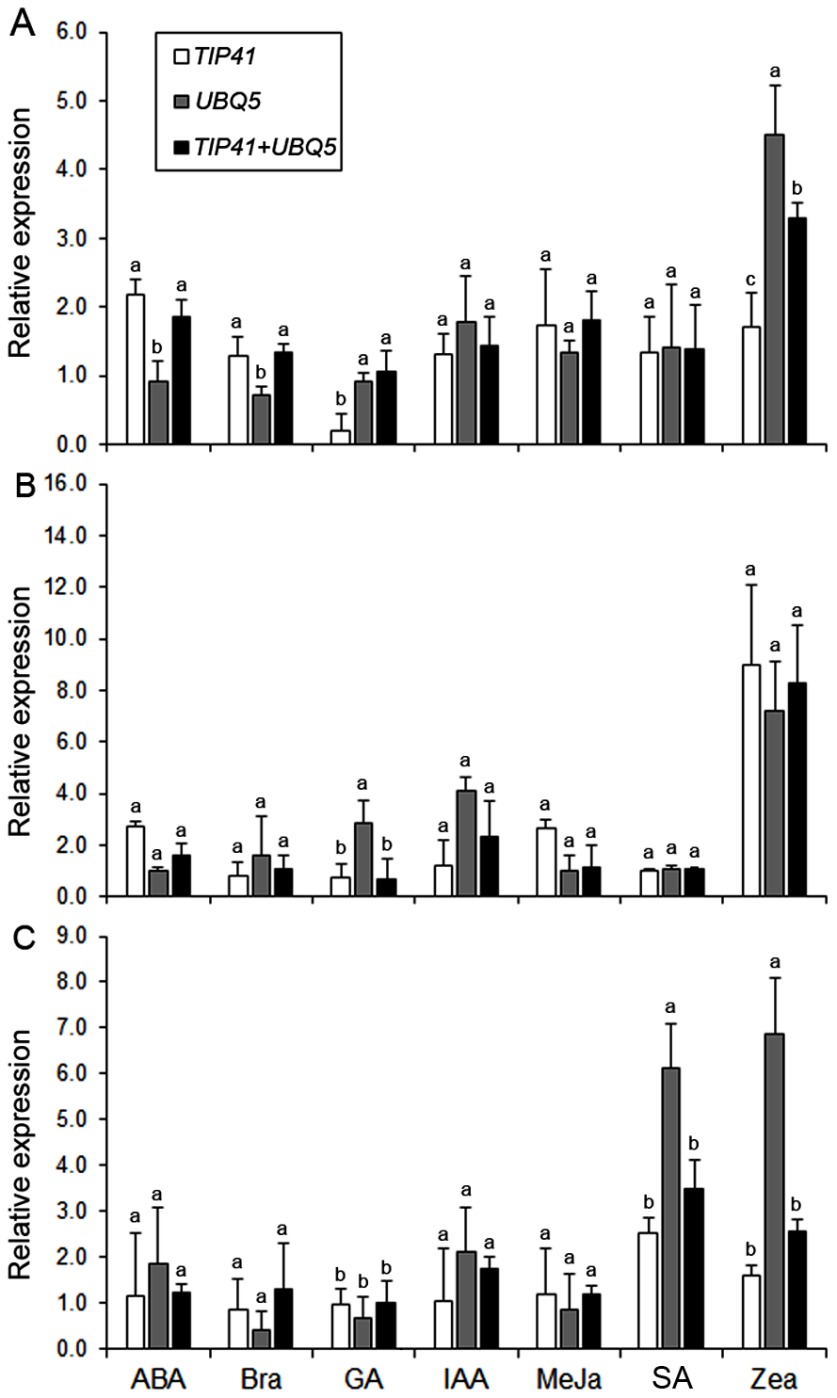
Validation of *ERF* gene expression after normalization using optimal number of control reference genes in hormone treated samples from genotypes (A) ICMR01004, (B) IPCI1466 and (C) IP300088. Data are presented as mean relative expression with SD from three biological replicates after normalization using the best combination of reference genes recommended by geNorm and RefFinder (see Table 8) for hormone treatments. ABA (abscisic acid), Bra (brassinolide), GA (gibberellic acid), IAA (indole-3-acetic acid), MeJa (methyl jasmonate), SA (salicylic acid) and Zea (zeatin) treatments of 15DPG plants. Different letters on the bars indicate significant differences at the *P*≤0.05 level as tested by Tukey's Range Test.

**Figure 4:**
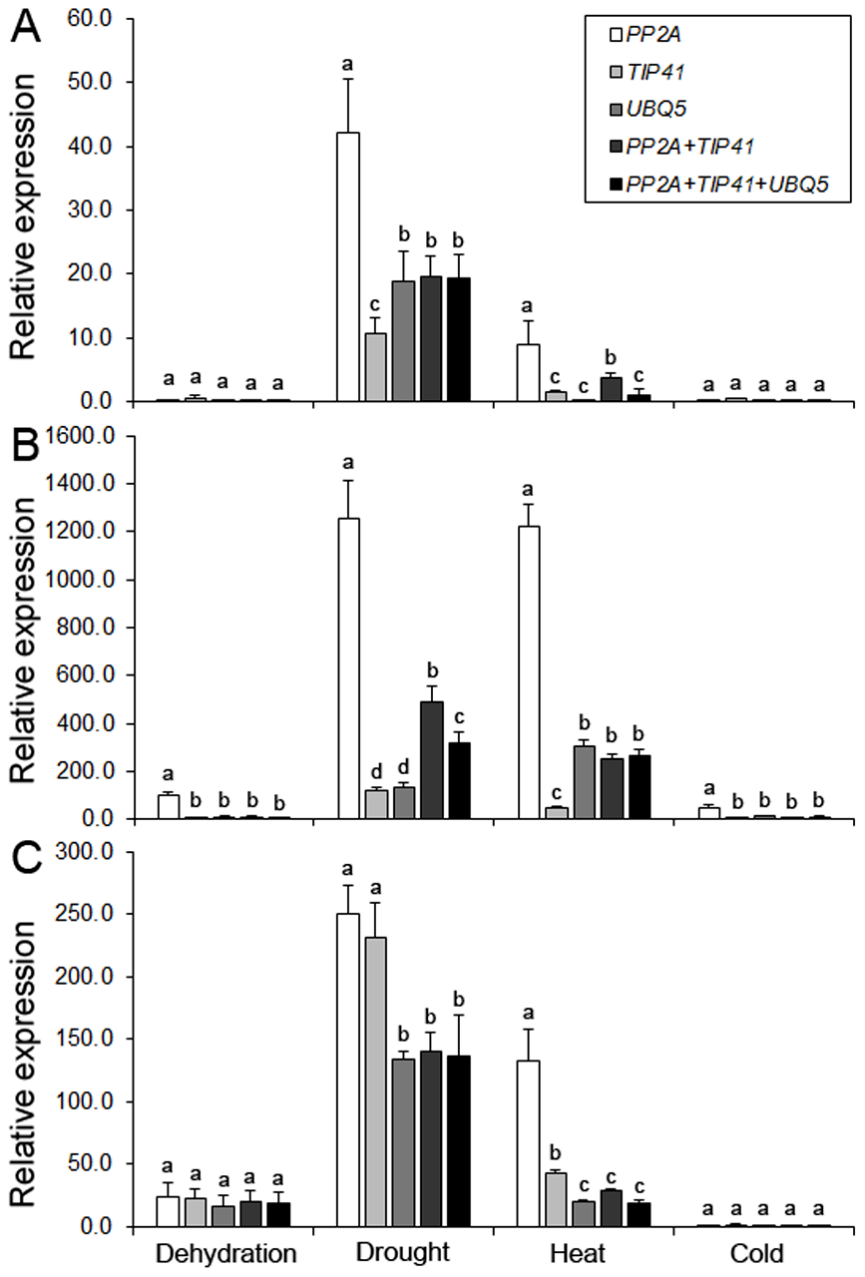
Validation of *DREB* gene expression after normalization using optimal number of control reference genes in genotypes (A) ICMR01004, (B) IPCI1466 and (C) IP300088 subjected to abiotic stress conditions. Results are presented as mean relative expression with SD from three biological replicates after normalization using the best combination of reference genes recommended by geNorm and RefFinder (see Table 8) for abiotic stress conditions. Dehydration (mannitol), drought (no water), heat (42°C) and cold (4°C) stresses are presented. Different letters on the bars indicate significant differences at the *P*≤0.05 level as tested by Tukey's Range Test.

In order to validate the selection of the best reference genes for accurate normalization of gene expression, we chose *PEPC* (phosphoenolpyruvate carboxylase), *ERF* (ethylene response factor) and *DREB* (dehydration responsive element binding) genes to determine the relative transcript levels using qRT-PCR (Table S2). We monitored the expression of *PEPC*, an essential gene for C4 photosynthesis, in developmental tissue samples, whereas the expression pattern of two transcription factors, *ERF* and *DREB*, known to be regulated during abiotic and biotic stresses, were examined in hormone treated and abiotic stressed samples. Relative transcript levels of these genes were calculated after normalizing with the best ranked candidate reference genes as determined by geNorm and recommended by RefFinder (Table 8). Transcript abundance of *PEPC* when normalized using single top ranked reference genes, *PP2A*, *TIP41* and *UBC2*, revealed bias effect on the relative expression patterns (Figure 2). Furthermore, transcript normalization using a combination of two (*PP2A*+*TIP41*) and three (*PP2A*+*TIP41*+*UBC2*) reference genes showed much stable and constant expression profiles across tissues (Figure 2). Similarly, relative expression patterns of *ERF* and *DREB* in hormone treatments and abiotic stress conditions were affected by the selection of the reference gene or combination of genes, respectively (Figures 3–4). As predicted, a strong bias in the relative expression pattern of *PEPC, ERF* and *DREB* was obtained when the least stable gene was used for normalization. Incorporation of *TIP41* and *UBC2* or *UBQ5* during expression analyses neutralized the unwanted changes of transcript abundance to allow accurate normalization of *PEPC*, *ERF* and *DREB*. Overall expression of *PEPC* was significantly high in flag leaf and sheath as compared to nodal tissues of pearl millet genotypes (Figure 2). In the hormone treatments experimental set, Zea enhanced 2-fold expression of *ERF* in pearl millet genotype ICMT01004 and IPCI1466 compared to other hormones tested (Figure 3). The expression of *DREB* was up-regulated during drought followed by heat stresses in all the three genotypes (Figure 4). Genotypes showed differential expression patterns of these genes as well (Figures 2–4).

**Figure 5:**
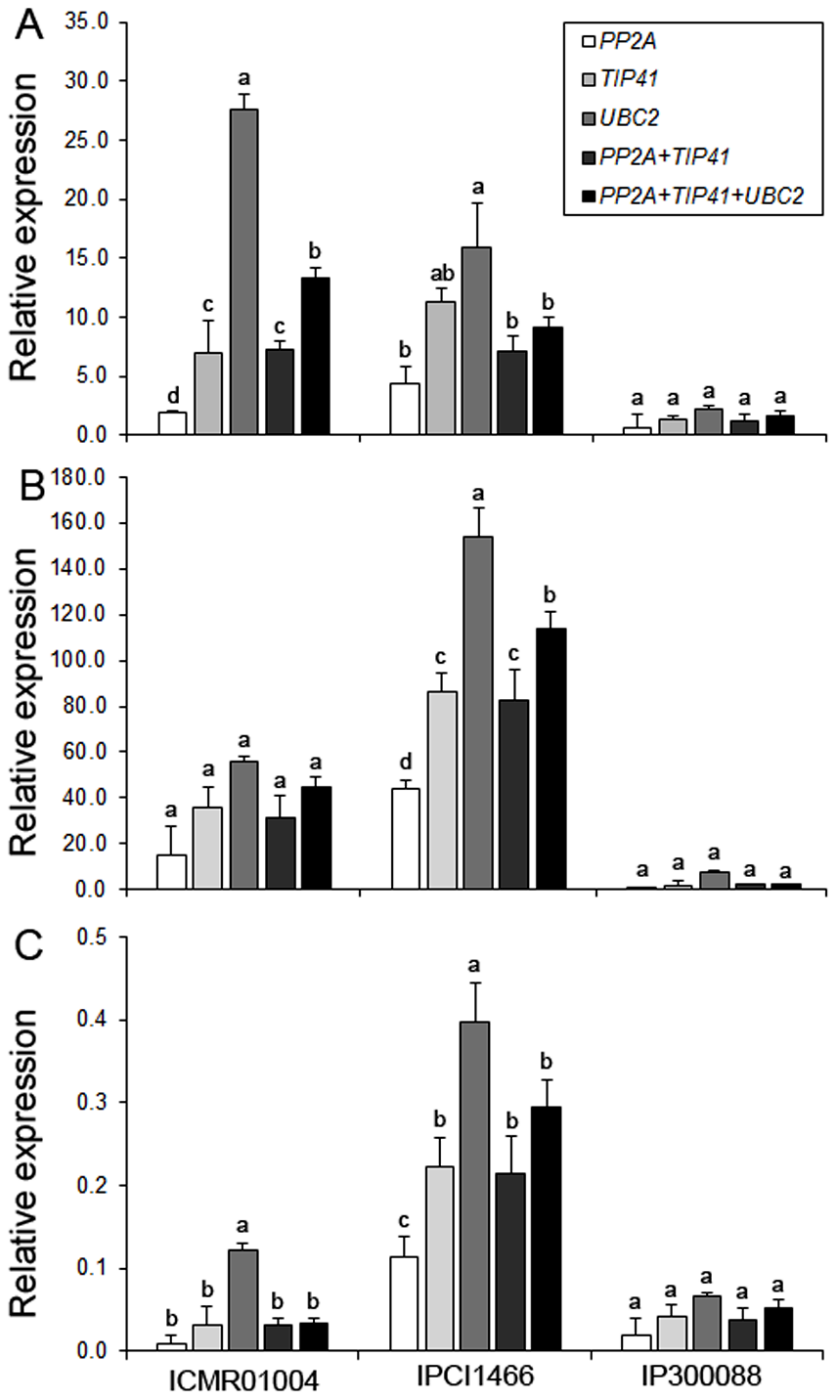
Validation of expression of *gus, gfp and hpt* transgenes using optimal number of control reference genes in hygromycin resistant calli from genotypes (A) ICMR01004, (B) IPCI1466 and (C) IP300088 after 30 days post particle bombardment-mediated transformation using pCAMBIA1201 and pCAMBIA1302, respectively. Results are presented as mean relative expression with SD from three biological replicates after normalization using the best combination of reference genes recommended by geNorm and RefFinder (see Table 8) for developmental tissue samples. Different letters on the bars indicate significant differences at the *P*≤0.05 level as tested by Tukey's Range Test.

We also monitored the transcript abundance pattern of *β-glucuronidase* (*gus*), *green fluorescent protein* (*gfp*) and *hygromycin phosphotransferase* (*hpt*) expressing transgenes in transgenic pearl millet calli. Calli of three pearl millet genotypes were bombarded with *CaMV35S::gus* (pCAMBIA1201) and *CaMV35S::gfp* (pCAMBIA1302) constructs and transient expression of both *gus* and *gfp* reporter genes were visualized after 5 days (Figure S3). Expressions of *gus*, *gfp* and *hpt* genes were examined in transformed calli selected on hygromycin (30 mg/l) after 30 days post bombardment using qRT-PCR. Normalization with the recommended reference genes (*PP2A*, *TIP41* and *UBC2*) showed similar effects on the relative expression patterns of *gus*, *gfp* and *hpt* transgenes in the calli of all three genotypes (Figure 5) as observed for *PEPC* in leaves (Figure 2), whereas the combination of the two (*PP2A*+*TIP41*) and the three (*PP2A*+*TIP41*+*UBC2*) reference genes exhibited more reliable transcript quantification. In general, expression analyses revealed that relative quantification of all three transgenes were higher in pearl millet genotype ICMT01004 and IPCI1466 compared to IP300088 (Figure 5).

## Discussion

Transcriptome changes occurring during developmental processes and/or adverse environmental conditions are experiencing a growing research interest to understand the gene regulatory networks that control agronomically and economically important traits e.g. enhanced crop yield and biomass production under high atmospheric CO_2_ or abiotic stress in the Panicoideae grasses including pearl millet. Transcriptomics data from microarray and next generation sequencing analyses should be validated using qRT-PCR [41]. QRT-PCR provides a useful tool to study transcriptome changes in pearl millet because no genome sequence or microarray chip is available. Moreover, reliable transcript measurements using qRT-PCR analysis require accurate normalization against an appropriate internal control reference gene [9], [28]. Normalization is important to adjust the variation introduced by various steps involved in the qRT-PCR such as quantity and quality of RNA samples, cDNAs, fluorescent fluctuations, sample-to-sample and/or well-to-well volume variations [10]. Therefore, pearl millet requires an assessment of appropriate reference genes for accurate transcript normalization in gene expression studies using qRT-PCR.

In this study, we demonstrated a comprehensive analysis of 18 potential candidate reference genes which included both traditional housekeeping genes like *ACT*, *eEF1α*, *GAPDH*, *TUA*, *UBC* and *UBQ5* and new candidate reference genes e.g. *PEPKR*, *PP2A*, *TIP41* on 234 samples from developmental tissues, hormone treatments and abiotic stress conditions of three pearl millet genotypes. We carried out simple total RNA extraction protocols using the guanidinium thiocyanate-based kit [42], which yielded acceptable RNA quality and quantity from all samples including roots and seeds of three pearl millet genotypes as mentioned by the golden rules of qRT-PCR [11]. Previously published protocols using same guanidinium thiocyanate-based kit demonstrated satisfactory amount of high quality RNA from rice [43]. Since DNA contamination can result in inaccurate quantification of RNA abundance [44], we conducted a second gDNA wipeout reaction on the isolated RNAs after on-column DNase treatment following manufacturer recommended protocol to completely eliminate the detectable genomic DNA contamination as verified by qRT-PCR for absence of any non-specific amplification. We primed cDNA synthesis using an optimized blend of oligo-dT and random primers to preferentially amplify the lowly abundant transcripts such as *CYC* in this study. In support of our finding, a weak expression of *CYC* was observed in rice [43]. However, the abundance of *25S rRNA* in different tissue, physiological conditions and pearl millet genotypes suggested the use of random hexamers to prime the reverse transcription reaction in our study.

We performed the two-step qRT-PCR method to reduce the unwanted primer dimer formation using SYBR Green detection dye [8]. This method was also followed for the large scale expression profiling of transcription factors in rice [43]. Specific amplification with expected amplicon size of each primer pair from the RT-PCR was confirmed by agarose gel electrophoresis (Figure S1). In addition, the single peak melting curves in the qRT-PCR with no amplicon peak in the NTCs proved the absence of primer dimers or non-specific products (Figure S2). The PCR efficiency of each primer pair was calculated from the raw amplification curves (absolute fluorescence data) captured during the exponential phase of amplification of each qRT-PCR reaction using LinRegPCR [37]. Except for *CYC* and *25S rRNA*, which showed an average efficiency of 1.87±0.03 and 1.86±0.04, all the candidate reference genes exhibited mean efficiency values greater than 1.90 (Table S1) suggesting specific transcripts being amplified at least at 90% efficiency per cycle in the qRT-PCR reactions [45]. An identical range of PCR efficiencies were reported for many orthologous of selected candidate reference genes from Arabidopsis [12], rice [43] and common bean [46]. In this study the average Ct (Tables 1–2 and S3–S5) values of candidate reference genes varied within the recommended range of 22.8±3.1 to 31.5±3.0 by qRT-PCR [47], except for *25S rRNA* which showed Ct of 9.1±1.8 (Table 1). In support of our results, a low Ct (average Ct value of 8) of *25S rRNA* gene was also observed in rice [13]. The ΔCt was calculated using the previously published method [39] and the precision of the assay was assessed using the CV. In general, our candidate reference genes showed CV<5% of Ct values, suggesting higher stability in expression levels under all experimental conditions. Therefore, our data demonstrated that the selected reference genes in this study are potential candidates for accurate normalization of gene expression by qRT-PCR after proper validation. In conjunction of our study, low CV<5% of Cq values of reference genes under abiotic stress conditions in common bean was also reported [46].

It has been suggested that the selection of optimal number of reference genes must be experimentally determined [48]. However, no single reference gene was found to have a stable expression under different experimental conditions [10], [33] and nor a single method is enough to test for the stability of the candidate reference genes [31], [33]. We used the algorithms executed by six different programs for proper stability ranking of the candidate reference genes. The SI [28] and ΔCt [29] methods calculate the variation of Ct and ΔCt values in pairwise genes, whereas BestKeeper estimates the variation in Ct values and reference genes showing SD<1 are considered the most stable [30]. However, the NormFinder [32] and geNorm [31] statistical algorithms allowed us to determine the stability ranking by calculating the SV and MV of each reference gene, respectively (Tables 3–7). In our study geNorm analyses revealed MV<1.5 for most of the genes under different experimental conditions (Tables 3–7), suggesting the potential stability of reference genes [31]. However, in the total experimental set *PEPKR* was the first ranked candidate gene by SI and BestKeeper, but ranked third by geNorm (Table 3); this could be due to the sensitivity of geNorm to the co-regulation of genes with similar expression patterns. In addition, geNorm is less affected by expression intensity of the reference genes [49] and allowed us to determine the optimal number of genes required to accurately normalize qRT-PCR data based on the V values [31]. We applied RefFinder [33] for recommended comprehensive ranking by combining all five above programs. Earlier reports on bamboo [17], strawberry [49] and leafy spurge [50] showed that these computational programs did not place the top ranked genes in identical order. According to our analysis, the six statistical programs ranked the candidate reference genes in various orders from best to worst, which could be due to different algorithm used by each program. Overall, new reference genes ranked better than the traditional housekeeping genes by most of the programs (Tables 3–8). Normalization using multiple reference genes is critical not only to obtain reliable gene expression results since normalization using single gene can be erroneous [9], but it also evaluates the expression stability of the selected reference genes during qRT-PCR. The geNorm analyses allowed us to identify optimal number of reference genes (Table 8) required for accurate normalization by calculating the V values at the suggested cut-off range of 0.15 [31].

In this study all the six computational methods suggested that *PP2A*, *TIP41*, *UBC2*, *UBQ5* and *ACT* are the top 5 superior reference genes for accurate transcript normalization in pearl millet under different experimental conditions (Table 8). None of the traditional housekeeping genes qualified as the best reference gene for transcript normalization in total tissue across all the five experimental sets of pearl millet. Moreover, only *UBQ5* and *ACT* were found to be suitable for hormone treated, stress conditions and genotypes of pearl millet (Table 8), respectively. This is because expression stability of many housekeeping genes vary considerably owning to their involvement in the cellular metabolism and functions [26]. In accordance to our study, *ACT* was one of the best reference genes in foxtail millet [20]. In addition, *ACT* was shown be a good candidate reference gene for normalization of transcript data in rice [43] and strawberry [49]. Moreover, *UBQ* was found to be a suitable reference gene in mustard [21], poplar [28] and rice [13]. In the current study, *18S rRNA*, *25S rRNA* and *SAMDc* were consistently categorized as unsuitable, perhaps due to their inconsistency in gene expression by all the six programs (Tables 3–7), thereby rendering them inappropriate to use as reference gene. Similarly, poor stability of *18S rRNA* under abiotic stress conditions was reported in foxtail millet [20]. In conjunction with rice the high expression of *25S rRNA* in this study makes it inappropriate for normalization of weakly expressed genes [13]. We observed significant variation of *SAMDc* expression pattern, which has been shown recently to be a poor reference gene in switchgrass [25]. The *CYC*, *eEF1α* and *eIF4a* were listed as variable genes in many studies [24], [43], thereby limiting their use as reference genes in pearl millet as well. We found *GAPDH* as an inappropriate reference gene, which was also ranked unsuitable for bamboo [17], brachypodium [14] and rice [13]. In our study, another traditional housekeeping gene *UBC2* ranked the third best reference genes after two novel candidate reference genes, *PP2A* and *TIP41* for normalization in developmental tissue samples. The UBC encodes an ubiquitin-conjugating enzyme E2 involved in protein degradation through ubiquitination reactions and performed best among the three traditional housekeeping genes in leafy spurge [50]. However, in the current study two novel candidate genes, *PP2A* and *TIP41* resulted as superior reference genes compared to traditional housekeeping genes tested under different experimental conditions. This finding is in agreement with previous reports where *PP2A* and *TIP41* combination was most suitable for abiotic stress conditions in caragana [27]. Recent reports demonstrated that *PP2A* and *TIP41* were the most recommended stable reference genes for transcript normalization in tissue samples of numerous plant species [17], [19], [27].

The suitability of these reference genes to conduct transcriptomics studies was assessed by monitoring the expression profiles of three endogenous genes and transgenes in both untransformed and genetically transformed pearl millet tissues. The *PEPC* encodes a ubiquitous cytosolic enzyme in higher plants which catalyzes the irreversible carboxylation of phosphoenolpyruvate (PEP) to oxaloacetate (OAA), a four carbon compound, in the initial fixation of atmospheric CO_2_ during C4 photosynthesis [51]. We noticed that transcript levels of *PEPC* were high in the flag leaf compared to nodal tissue in all the pearl millet genotypes studied (Figure 2). The ERF and DREB are AP2 binding transcription factors which regulate plant responses to several environmental stress conditions [52] and up-regulated under abiotic stresses [52] and hormone signaling [53], respectively. Transcript abundance of *ERF* illustrated differential expression pattern after accurate quantification using *TIP41* and *UBQ5* under different hormone stimuli conditions (Figure 3). Currently, several reports have validated the optimum relative expression of *DREB* using appropriate reference genes under abiotic stress conditions [21], [27]. In agreement with previous reports, we found *DREB* expression was up-regulated many fold in drought and heat stress conditions after accurate normalization using combination of reference genes (Figure 4). In addition, we provided evidence that these set of reference genes are also useful for transcript quantification in transformed pearl millet tissues, while incorporation of multiple reference genes provides the most reliable expression pattern after precise normalization.

## Conclusions

To the best of our knowledge this is the first comprehensive assessment of appropriate reference genes for accurate transcript normalization using qRT-PCR analyses in pearl millet. Stability ranking using computer based Stability Index, ΔCt, BestKeeper, NormFinder, geNorm and RefFinder programs recommended *TIP41*, *PP2A*, *UBC2*, *UBQ5* and *ACT* as the best reference genes out of 18 potential candidate genes tested on different developmental and experimental conditions. This work will facilitate the developmental gene expression studies on C4 photosynthesis and hormone cross-talk during abiotic stress conditions in pearl millet, a crop with limited genomic and transcriptomics information, and also benefit the scientific community for conducting experiments on related bioenergy crop species.

## Supporting Information

Figure S1 **Reverse transcription (RT)-PCR conformation of individual candidate reference gene showing specific amplification of the expected amplicon size from each primer pair in 3% (w/v) agarose gel.** cDNAs prepared from RNA samples isolated from leaves of 30D old plants from three biological replicates were pooled together and PCR reactions were conducted using primer pair specific for each candidate reference gene. Lane name corresponds to each reference gene used for RT-PCR. M1 and M2 are 50 base pair (bp) and 100 bp DNA ladder, respectively.
(TIF)

Figure S2 **Dissociation curve analyses for conformation of specific real-time PCR amplification with single peak for each primer pair.** cDNAs were prepared from RNA samples isolated from flag leaves in three biological replicates and melt curves generated after qRT-PCR using primer pair specific for each gene with no template controls (NTC) are presented.
(TIF)

Figure S3 **Expression of reporter genes in particle bombarded pearl millet genotype ICMR01004 calli.** (A) *gus* reporter gene expression in calli bombarded with pCAMBIA1201 plasmid, (B) *gfp* reporter gene expression in calli after bombardment with pCAMBIA1302 plasmid. Both the reporter genes were driven by CaMV35S promoter and the expression was monitored after 5 days post bombardment.
(TIF)

Table S1 Primer sequences of candidate reference genes used for qRT-PCR.
(DOCX)

Table S2 Information of selected endogenous genes and transgenes with primer sequences for validation of accurate normalization using suitable reference genes.
(DOCX)

Table S3 Distribution of the Ct values of each candidate reference genes across the developmental tissue samples of pearl millet.
(DOCX)

Table S4 Distribution of Ct values of each candidate reference genes in pearl millet samples subjected to hormone treatments.
(DOCX)

Table S5 Distribution of Ct values of each candidate reference genes in pearl millet samples subjected to abiotic stress conditions.
(DOCX)
