# Supplementary figures and images for: Assessing Reference Genes for Accurate Transcript Normalization Using Quantitative Real-Time PCR in Pearl Millet [Pennisetum glaucum (L.) R. Br.]
Source: PLoS One. 2014 Aug 29;9(8):e106308. doi: 10.1371/journal.pone.0106308 (PMC4149553; doi:10.1371/journal.pone.0106308)

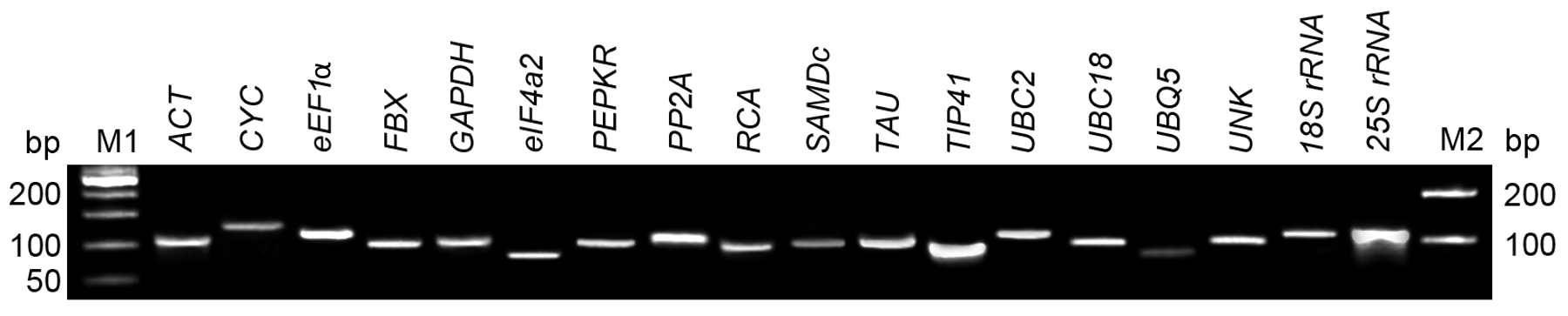

Supplement: Figure S1 — Reverse transcription (RT)-PCR conformation of individual candidate reference gene showing specific amplification of the expected amplicon size from each primer pair in 3% (w/v) agarose gel. cDNAs prepared from RNA samples isolated from leaves of 30D old plants from three biological replicates were pooled together and PCR reactions were conducted using primer pair specific for each candidate reference gene. Lane name corresponds to each reference gene used for RT-PCR. M1 and M2 are 50 base pair (bp) and 100 bp DNA ladder, respectively. (TIF) [file pone.0106308.s001.tif]

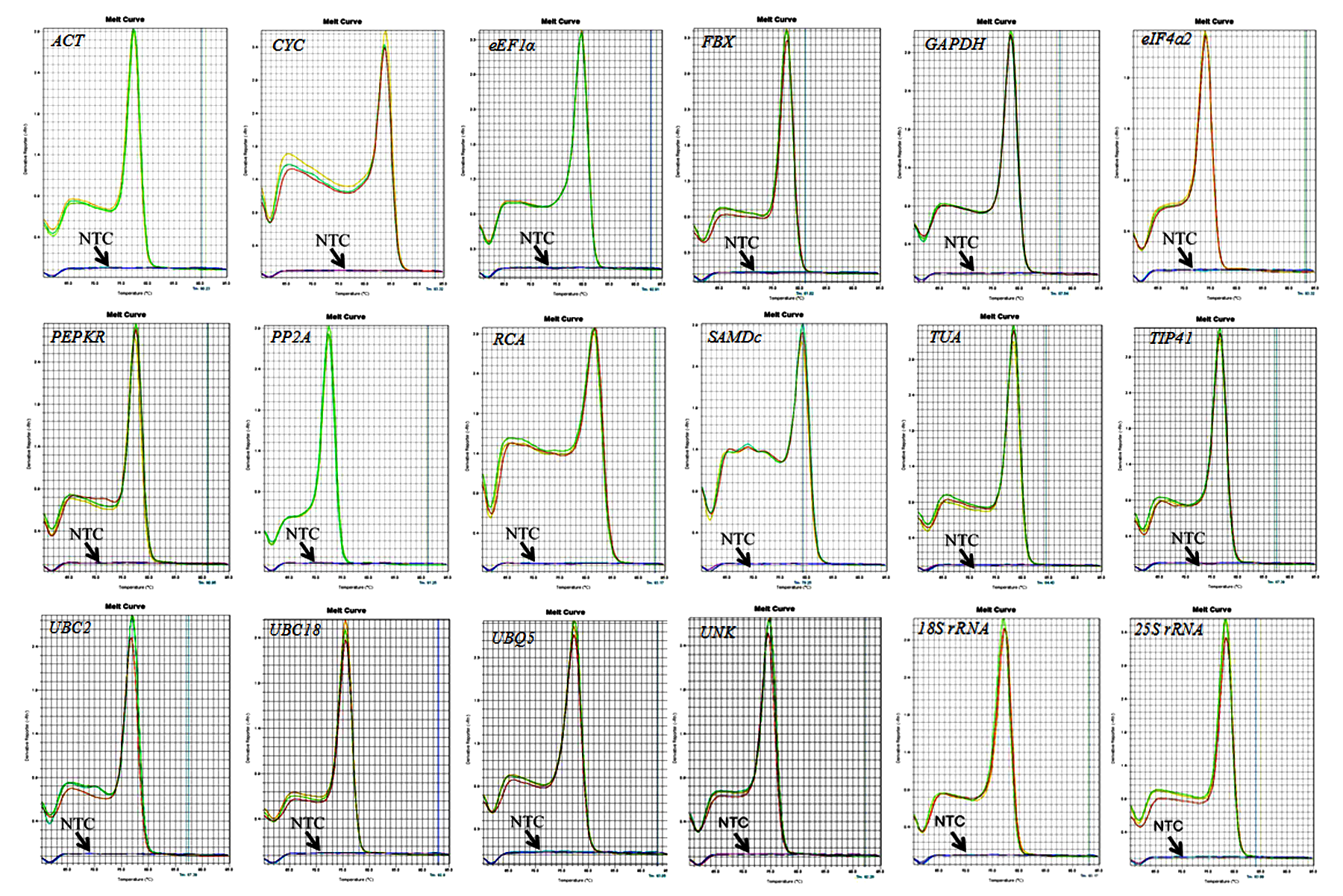

Supplement: Figure S2 — Dissociation curve analyses for conformation of specific real-time PCR amplification with single peak for each primer pair. cDNAs were prepared from RNA samples isolated from flag leaves in three biological replicates and melt curves generated after qRT-PCR using primer pair specific for each gene with no template controls (NTC) are presented. (TIF) [file pone.0106308.s002.tif]

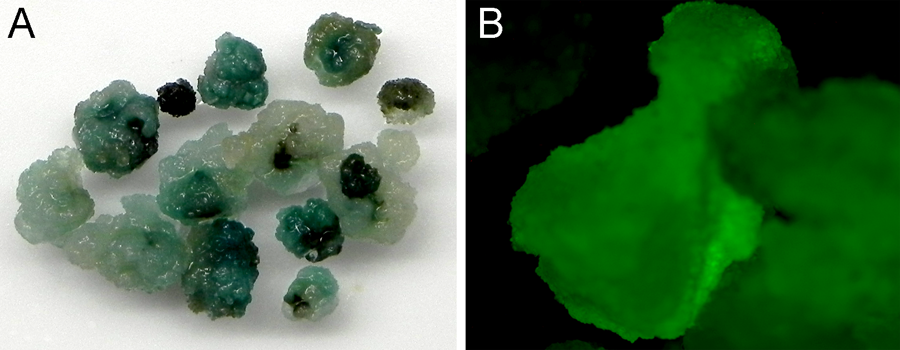

Supplement: Figure S3 — Expression of reporter genes in particle bombarded pearl millet genotype ICMR01004 calli. (A) gus reporter gene expression in calli bombarded with pCAMBIA1201 plasmid, (B) gfp reporter gene expression in calli after bombardment with pCAMBIA1302 plasmid. Both the reporter genes were driven by CaMV35S promoter and the expression was monitored after 5 days post bombardment. (TIF) [file pone.0106308.s003.tif]
