# Supplementary material for: Assessing Reference Genes for Accurate Transcript Normalization Using Quantitative Real-Time PCR in Pearl Millet [Pennisetum glaucum (L.) R. Br.]
Source: PLoS One. 2014 Aug 29;9(8):e106308. doi: 10.1371/journal.pone.0106308 (PMC4149553; doi:10.1371/journal.pone.0106308)
Supplement: Table S1 — Primer sequences of candidate reference genes used for qRT-PCR. (DOCX) [file pone.0106308.s004.docx]

**Table S1.** Primer sequences of candidate reference genes used for qRT-PCR.

| Genes | Forward primer | Forward sequence | Reverse primer | Reverse sequence | Size (bp) | PCR efficiency (*E*) | *R^2^* |
| --- | --- | --- | --- | --- | --- | --- | --- |
| *ACT* | *ACT*_qF896 | TGCTCAGTGGAGGATCTACTAT | *ACT*_qR1004 | CTGGTGGTGCAATCACTTTAAC | 108 | 1.91±0.02 | 0.92 |
| *CYC* | *CYC*_qF313 | TTTCGCTCTGACAGACCTTTAG | *CYC*_qR451 | TGGCAGTAGTAGTAGGGAGAAG | 138 | 1.87±0.03 | 0.94 |
| *eEF1α* | *eEF1a*_qF226 | GGAAGTTTGAGACCACCAAGTA | *EF1a*_qR355 | CAGTGGTAGGGTCAATGATGAG | 129 | 1.90±0.04 | 0.93 |
| *FBX* | *FBX*_qF911 | GTTCCCTGCTGGATGGTTAT | *FBX*_qR1017 | AGCTCCCAGATCCTCATACT | 106 | 1.90±0.03 | 0.93 |
| *GAPDH* | *GAPDH*_qF692 | CTGGTATGTCCTTCCGTGTTC | *GAPDH*_qR780 | GCAGCCTTGATAGCCTTCTTA | 109 | 1.90±0.01 | 0.95 |
| *eIF4a2* | *eIF4a2*_qF560 | TTGTCCTGGATGAAGCTGATG | *eIF4a2*_qR628 | CCTGAATCTTGGATGGGAGAAG | 90 | 1.90±0.02 | 0.91 |
| *PEPKR* | *PEPKR*_qF651 | TGAAGCTGCCGATGTATGG | *PEPKR*_qR749 | GCAGCTCTGTGGACCTTATAC | 119 | 1.91±0.02 | 0.91 |
| *PP2A* | *PP2A*_qF345 | TGAGAGCAGACAAATCACTCAA | *PP2A*_qF444 | AAGAGCTGTGAGAGGCAAATAA | 121 | 1.92±0.03 | 0.92 |
| *RCA* | *RCA*_qF490 | GACATCATCAAGAAGGGCAAGA | *RCA*_qR575 | CCATCTGGTTGTTGACGGTAT | 106 | 1.90±0.01 | 0.95 |
| *SAMDc* | *SAMDc*_qF885 | GTTCTCTGTTGCTGTGACTATCT | *SAMDc*_qR970 | CTCCTGCTCTACCATGTTGTT | 106 | 1.91±0.03 | 0.96 |
| *TUA* | *TUA*_qF463 | GAGCGTCTGTCTGTTGACTATG | *TUA*_qR551 | GTGGACAGGACACTGTTGTATG | 110 | 1.90±0.02 | 0.89 |
| *TIP41* | *TIP41*_qF405 | GGTTCTGAACTCAGGCACTAC | *TIP41*_qR480 | GAAAGGGCAACAAGGTCAATC | 96 | 1.91±0.02 | 0.94 |
| *UBC2* | *UBC2*_qF302 | CATGGACCCTTCAGTCTGTTT | *UBC2*_qR395 | CCTCGGATATCACCCGATCTTA | 115 | 1.91±0.02 | 0.93 |
| *UBC18* | *UBC18*_qF285 | CGGCCACATCTGCTTAGATATT | *UBC18*_qR365 | GCTGGCGAACTAGACAACATAG | 102 | 1.90±0.02 | 0.93 |
| *UBQ5* | *UBQ5*_qF252 | GTACACCAAGCCCAAGAAGA | *UBQ5*_qR310 | GTCGTCGACCTTGTAGAACTG | 79 | 1.91±0.03 | 0.91 |
| *UNK* | *UNK*_qF448 | CACCATATGCTGTCGCTTATTTC | *UNK*_qR534 | TGATTCCAGGTGTGGTTGTT | 106 | 1.90±0.04 | 0.93 |
| *18S rRNA* | *18SrRNA*_qF773 | CCATGCAGTCTCTCCAACTTAC | *18SRNA*_qR884 | CAGTCTCCATGCTCAGATCTTC | 111 | 1.91±0.02 | 0.95 |
| *25S rRNA* | *25SrRNA_*qR96 | CGTGCCGCGATAGTAATTCA | *25SrRNA*_qR188 | GTCATAATCCGACACACGGTAG | 114 | 1.86±0.04 | 0.95 |

Number in the forward (F) and reverse (R) primer represents the position on the RNA coding sequence (CDS). Mean PCR efficiency (*E*) ±SD including the mean regression coefficient (*R^2^*) were determined from three biological replicates of all samples using LinRegPCR [[37](#_ENREF_37)]. bp, base pair.
