# Supplementary material for: Assessing Reference Genes for Accurate Transcript Normalization Using Quantitative Real-Time PCR in Pearl Millet [Pennisetum glaucum (L.) R. Br.]
Source: PLoS One. 2014 Aug 29;9(8):e106308. doi: 10.1371/journal.pone.0106308 (PMC4149553; doi:10.1371/journal.pone.0106308)
Supplement: Table S2 — Information of selected endogenous genes and transgenes with primer sequences for validation of accurate normalization using suitable reference genes. (DOCX) [file pone.0106308.s005.docx]

**Table S2.** Information of selected endogenous genes and transgenes with primer sequences for validation of accurate normalization using suitable reference genes.

| Genes | Description | Arabidopsis | Rice | Millet^a^ | Primers | Sequence (5’-3’) | Size (bp) |
| --- | --- | --- | --- | --- | --- | --- | --- |
| *DREB* | Dehydration responsive element binding protein | At4g25480 | LOC_Os04g48350 | Si011005m.g/  AY829439 | *DREB*_qF513 | CGGCGACTTCGCTTACTAC | 138 |
|  |  |  |  |  | *DREB*_qR632 | TCGTAGTCGTCCTCGATCC |  |
| *ERF* | Ethylene response factor | At3g23240 | LOC_Os03g64260 | Si038911m.g | *ERF1* qF696 | ATGCGTTGGCGTAGATGTAG | 115 |
|  |  |  |  |  | *ERF1_*qR811 | TCAGATGGAGTGGTGGCT |  |
| *PEPC* | Phosphoenolpyruvate carboxylase | At1g53310 | LOC_Os01g11054 | Si005789m.g/ HQ850700 | *PEPC*_qF849 | TCGGGATTGATGAGCGTTTC | 97 |
|  |  |  |  |  | *PEPC*_qR9 | TCTGGCGTAACTCTTGGATTTC |  |
| *gus* | *β-glucuronidase* reporter gene from pCAMBIA1201 | NA | NA | AF234293 | *GUS_qF1519* | GAATACGGCGTGGATACGTTAG | 106 |
|  |  |  |  |  | *GUS_qR1625* | GATCAAAGACGCGGTGATACA |  |
| *gfp* | *Green fluorescent protein* reporter gene from pCAMBIA1302 | NA | NA | AAF65344 | *GFP_qF591* | TCCACACAATCTGCCCTTTC | 124 |
|  |  |  |  |  | *GFP_qR715* | GGTGGTGGCTAGCTTTGTATAG |  |
| *hpt* | *Hygromycin phosphotransferase*  selective marker gene | NA | NA | AF234293 | *HPT_qF820* | CGATGCAAAGTGCCGATAAAC | 102 |
|  |  |  |  |  | *HPT_qR922* | GCTTTCAGCTTCGATGTAGGA |  |

Number in the forward (F) and reverse (R) primer represents the position on the RNA coding sequence (CDS). ^a^ Locus identifiers of selected candidate reference genes for foxtail millet and/or GenBank accession numbers for pearl millet with orthologous from Arabidopsis and rice are listed . NA, not applicable; bp, base pair.
