# Supplementary material for: Assessing Reference Genes for Accurate Transcript Normalization Using Quantitative Real-Time PCR in Pearl Millet [Pennisetum glaucum (L.) R. Br.]
Source: PLoS One. 2014 Aug 29;9(8):e106308. doi: 10.1371/journal.pone.0106308 (PMC4149553; doi:10.1371/journal.pone.0106308)
Supplement: Table S3 — Distribution of the Ct values of each candidate reference genes across the developmental tissue samples of pearl millet. (DOCX) [file pone.0106308.s006.docx]

**Table S3.** Distribution of the Ct values of each candidate reference genes across the developmental tissue samples of pearl millet.

| Genes | Callus | Leaf 7D | Leaf 15D | Leaf 30D | Flag leaf | Sheath | Node | Internode | Panicle | Peduncle | Root | Seed |
| --- | --- | --- | --- | --- | --- | --- | --- | --- | --- | --- | --- | --- |
| *ACT* | 27.9±0.9 | 29.7±1.1 | 28.9±1.1 | 31.2±4.7 | 27.3±1.6 | 27.4±2.3 | 26.0±2.5 | 27.5±2.8 | 25.1±2.0 | 27.8±2.9 | 29.0±1.8 | 32.4±2.0 |
| *CYC* | 33.9±0.7 | 33.8±1.0 | 32.6±0.6 | 32.8±1.6 | 29.9±2.1 | 28.9±1.5 | 26.5±2.0 | 26.4±1.9 | 28.9±1.2 | 27.6±0.6 | 28.2±0.4 | 33.4±0.6 |
| *eEF1α* | 22.2±2.7 | 22.6±2.8 | 22.1±2.4 | 23.8±2.0 | 22.5±2.8 | 22.3±2.5 | 21.2±1.8 | 22.4±2.3 | 21.4±2.6 | 22.9±2.2 | 22.2±3.0 | 30.1±4.1 |
| *FBX* | 21.9±1.5 | 25.6±0.7 | 25.5±1.8 | 26.3±1.6 | 26.8±1.6 | 26.0±2.6 | 22.5±0.8 | 23.7±0.9 | 22.0±0.8 | 26.3±2.0 | 23.8±2.2 | 33.6±1.1 |
| *GAPDH* | 22.2±1.2 | 23.1±1.2 | 22.4±0.6 | 26.0±4.3 | 21.6±1.9 | 21.7±1.9 | 21.9±3.3 | 24.3±4.3 | 20.8±2.0 | 21.0±1.8 | 22.6±0.4 | 30.3±0.7 |
| *eIF4a2* | 20.4±1.0 | 23.4±0.6 | 22.5±1.4 | 24.6±2.5 | 23.5±1.4 | 23.0±0.8 | 21.6±1.6 | 22.5±1.1 | 22.4±1.6 | 23.3±1.7 | 22.5±0.8 | 27.0±0.7 |
| *PEPKR* | 25.9±0.5 | 25.3±0.6 | 25.4±0.5 | 25.7±0.8 | 25.4±1.4 | 24.8±0.6 | 24.9±0.2 | 25.0±0.5 | 24.0±1.1 | 23.7±0.4 | 25.7±1.1 | 27.2±1.4 |
| *PP2A* | 25.9±2.3 | 26.2±1.9 | 25.1±0.6 | 27.1±2.3 | 24.7±1.1 | 24.7±0.6 | 23.9±0.1 | 24.7±0.2 | 23.7±0.5 | 24.3±0.7 | 26.0±2.0 | 32.6±2.4 |
| *RCA* | 30.5±1.1 | 24.0±0.4 | 23.9±0.1 | 24.3±0.9 | 23.3±2.1 | 22.4±1.4 | 24.2±1.5 | 23.1±1.4 | 23.8±0.6 | 22.8±1.3 | 30.7±1.2 | 29.9±1.2 |
| *SAMDc* | 27.0±6.8 | 28.7±4.4 | 23.2±4.2 | 24.8±5.9 | 25.8±6.4 | 25.8±6.0 | 24.9±5.5 | 25.3±4.9 | 25.5±6.4 | 25.7±5.9 | 27.1±4.7 | 33.4±0.3 |
| *TUA* | 21.1±0.5 | 22.8±0.1 | 21.9±0.3 | 25.1±1.0 | 25.3±1.4 | 22.9±1.9 | 20.1±0.4 | 20.4±1.2 | 20.2±0.3 | 20.6±2.9 | 22.5±0.9 | 32.3±1.3 |
| *TIP41* | 27.4±0.4 | 29.4±0.5 | 28.2±0.4 | 29.5±1.6 | 27.9±0.9 | 27.9±0.2 | 26.9±0.9 | 28.1±0.2 | 26.5±0.6 | 27.8±0.6 | 29.0±0.2 | 32.2±0.8 |
| *UBC2* | 30.3±3.3 | 30.3±1.7 | 29.6±2.3 | 31.2±4.3 | 28.7±3.0 | 28.4±3.2 | 28.4±2.9 | 28.8±3.1 | 28.0±2.8 | 28.6±2.7 | 30.0±2.2 | 32.0±0.6 |
| *UBC18* | 26.8±0.7 | 26.8±0.3 | 26.4±0.4 | 26.6±0.8 | 24.6±0.8 | 24.8±0.1 | 24.3±0.4 | 25.4±0.8 | 24.3±0.4 | 24.9±0.4 | 26.4±0.7 | 33.3±1.2 |
| *UBQ5* | 21.2±0.3 | 24.3±0.9 | 23.0±0.9 | 24.0±0.7 | 23.1±1.0 | 23.1±0.9 | 21.9±0.3 | 22.9±0.3 | 21.9±0.3 | 23.8±0.2 | 24.0±1.0 | 29.7±1.2 |
| *UNK* | 29.4±3.0 | 28.1±1.0 | 27.8±0.8 | 29.9±1.5 | 27.2±1.8 | 27.2±0.7 | 26.4±0.5 | 27.1±0.8 | 25.6±0.6 | 26.5±1.0 | 28.2±0.8 | 31.0±0.8 |
| *18S rRNA* | 19.0±3.6 | 22.8±4.5 | 21.8±5.6 | 23.7±6.0 | 22.3±4.5 | 21.2±5.4 | 20.5±5.8 | 21.5±4.9 | 21.1±5.2 | 22.0±5.4 | 22.0±4.5 | 28.5±1.5 |
| *25S rRNA* | 7.4±0.5 | 8.7±0.2 | 8.4±0.5 | 8.7±0.5 | 8.8±1.1 | 8.5±0.3 | 8.1±0.3 | 8.2±0.3 | 8.5±0.8 | 8.4±0.6 | 8.1±0.8 | 12.8±1.9 |

Data represent average Ct values±SD for each tissue sample from three pearl millet genotypes in three biological replicates.
