# Supplementary material for: Assessing Reference Genes for Accurate Transcript Normalization Using Quantitative Real-Time PCR in Pearl Millet [Pennisetum glaucum (L.) R. Br.]
Source: PLoS One. 2014 Aug 29;9(8):e106308. doi: 10.1371/journal.pone.0106308 (PMC4149553; doi:10.1371/journal.pone.0106308)
Supplement: Table S4 — Distribution of Ct values of each candidate reference genes in pearl millet samples subjected to hormone treatments. (DOCX) [file pone.0106308.s007.docx]

**Table S4**. Distribution of Ct values of each candidate reference genes in pearl millet samples subjected to hormone treatments.

| Genes | Control | Abscisic acid (ABA) | Brassinolide  (Bra) | Gibberellic acid (GA) | Indole-3-acetic acid (IAA) | Methyl jasmonate (MeJa) | Salicylic acid (SA) | Zeatin  (Zea) |
| --- | --- | --- | --- | --- | --- | --- | --- | --- |
| *ACT* | 28.9±1.1 | 26.8±1.0 | 27.6±1.2 | 27.8±2.4 | 27.1±2.3 | 26.9±1.2 | 29.2±1.7 | 28.2±1.6 |
| *CYC* | 32.6±0.6 | 33.3±1.0 | 33.1±0.7 | 33.2±0.8 | 33.7±2.3 | 33.1±1.0 | 33.6±0.5 | 33.5±0.5 |
| *eEF1α* | 22.1±2.4 | 26.4±0.8 | 25.8±1.3 | 25.7±1.5 | 25.6±1.5 | 25.9±1.3 | 25.5±1.1 | 25.4±1.3 |
| *FBX* | 22.5±1.4 | 22.9±0.7 | 22.6±1.2 | 22.6±1.2 | 22.0±1.6 | 22.7±1.3 | 23.2±3.4 | 22.2±0.8 |
| *GAPDH* | 25.5±1.8 | 26.0±1.2 | 24.0±1.0 | 23.7±1.3 | 24.3±0.6 | 24.5±0.5 | 27.8±5.2 | 23.9±0.4 |
| *eIF4a2* | 22.4±0.6 | 22.0±0.9 | 21.4±1.0 | 21.5±1.7 | 21.6±2.0 | 21.2±1.0 | 25.4±4.6 | 21.4±1.2 |
| *PEPKR* | 25.4±0.5 | 26.2±0.9 | 24.9±0.2 | 25.4±1.2 | 25.4±0.5 | 25.2±0.3 | 26.3±1.0 | 25.6±0.4 |
| *PP2A* | 25.1±0.6 | 25.4±2.0 | 24.7±1.1 | 24.9±1.6 | 24.4±0.1 | 24.5±0.9 | 29.2±1.6 | 24.7±1.2 |
| *RCA* | 23.9±0.1 | 24.8±0.4 | 23.4±0.7 | 24.1±1.4 | 23.8±1.4 | 24.0±0.7 | 30.2±1.5 | 24.4±0.9 |
| *SAMDc* | 23.2±4.2 | 25.4±3.6 | 25.0±4.2 | 25.5±4.2 | 25.2±5.3 | 24.9±3.8 | 27.1±6.8 | 25.3±4.3 |
| *TUA* | 21.9±0.3 | 22.1±1.2 | 21.0±0.5 | 21.5±1.0 | 21.0±1.5 | 21.2±0.4 | 25.9±5.7 | 21.5±0.8 |
| *TIP41* | 28.2±0.4 | 28.7±0.7 | 27.7±0.2 | 28.2±1.1 | 27.7±1.0 | 28.1±0.5 | 29.5±2.6 | 28.0±0.4 |
| *UBC2* | 29.6±2.3 | 29.0±1.5 | 28.7±2.0 | 28.7±2.1 | 28.4±2.7 | 28.7±2.1 | 32.0±4.7 | 28.7±1.9 |
| *UBC18* | 26.4±0.4 | 26.1±0.7 | 25.5±0.4 | 25.8±0.9 | 25.3±0.4 | 25.5±0.6 | 29.8±2.8 | 26.1±0.4 |
| *UBQ5* | 23.0±0.9 | 23.1±1.1 | 22.1±0.7 | 22.2±0.9 | 21.9±0.5 | 22.2±0.5 | 25.0±4.0 | 21.8±0.2 |
| *UNK* | 27.8±0.8 | 28.2±1.7 | 27.3±0.6 | 27.6±1.8 | 27.1±0.7 | 27.4±0.9 | 31.2±1.0 | 27.9±1.1 |
| *18S rRNA* | 21.8±5.6 | 27.6±0.5 | 26.9±0.9 | 27.3±1.7 | 27.1±2.2 | 27.0±1.2 | 23.0±7.9 | 27.2±1.5 |
| *25S rRNA* | 8.4±0.5 | 9.0±0.7 | 8.3±0.5 | 9.1±0.3 | 8.8±0.3 | 8.3±0.6 | 10.0±3.4 | 8.7±0.2 |

Data represent average Ct values±SD for each treatment from three pearl millet genotypes in three biological replicates.
