# Supplementary material for: Assessing Reference Genes for Accurate Transcript Normalization Using Quantitative Real-Time PCR in Pearl Millet [Pennisetum glaucum (L.) R. Br.]
Source: PLoS One. 2014 Aug 29;9(8):e106308. doi: 10.1371/journal.pone.0106308 (PMC4149553; doi:10.1371/journal.pone.0106308)
Supplement: Table S5 — Distribution of Ct values of each candidate reference genes in pearl millet samples subjected to abiotic stress conditions. (DOCX) [file pone.0106308.s008.docx]

**Table S5.** Distribution of Ct values of each candidate reference genes in pearl millet samples subjected to abiotic stress conditions.

| Genes | Control | Dehydration^a^ | Drought | Cold | Heat | Salinity |
| --- | --- | --- | --- | --- | --- | --- |
| *ACT* | 30.2±4.7 | 29.8±1.2 | 28.3±2.6 | 27.9±3.5 | 31.1±1.6 | 28.0±3.8 |
| *CYC* | 31.8±1.6 | 32.8±0.8 | 33.8±1.0 | 29.1±4.9 | 32.3±1.9 | 33.6±2.1 |
| *eEF1a* | 22.8±2.0 | 22.9±1.9 | 24.8±2.8 | 23.0±0.9 | 27.3±4.1 | 24.8±2.0 |
| *FBX* | 23.6±2.5 | 24.0±1.2 | 24.6±1.7 | 23.5±2.0 | 25.6±1.2 | 25.3±4.0 |
| *GAPDH* | 25.3±1.6 | 25.9±1.6 | 26.5±1.0 | 24.7±1.3 | 28.9±1.8 | 27.4±2.9 |
| *eIF4a2* | 25.0±4.3 | 23.4±1.2 | 22.9±2.2 | 21.2±2.7 | 26.7±1.8 | 23.7±3.9 |
| *PEPKR* | 24.7±0.8 | 24.6±0.7 | 26.6±1.8 | 24.8±0.7 | 26.1±1.7 | 28.3±4.0 |
| *PP2A* | 26.1±2.3 | 25.3±0.7 | 25.8±1.3 | 24.8±0.8 | 28.4±3.7 | 26.6±3.0 |
| *RCA* | 23.3±0.9 | 24.0±1.8 | 25.1±1.2 | 22.5±1.9 | 27.4±1.6 | 26.0±3.9 |
| *SAMDc* | 24.5±5.5 | 27.4±4.5 | 26.8±6.2 | 25.9±3.9 | 29.4±1.6 | 27.8±8.1 |
| *TUA* | 24.1±1.0 | 24.1±2.0 | 26.5±1.5 | 23.2±1.1 | 28.3±2.7 | 27.1±3.9 |
| *TIP41* | 28.5±1.6 | 28.5±0.8 | 29.2±1.4 | 28.3±0.9 | 29.7±1.6 | 30.0±2.7 |
| *UBC2* | 30.2±4.3 | 31.1±1.9 | 29.2±2.4 | 29.6±3.5 | 33.7±1.5 | 30.2±4.3 |
| *UBC18* | 25.6±0.8 | 26.3±0.5 | 26.2±1.3 | 25.4±1.1 | 30.0±3.5 | 26.4±1.4 |
| *UBQ5* | 23.0±0.7 | 23.4±1.2 | 24.4±2.0 | 24.0±1.3 | 26.3±3.1 | 24.8±1.5 |
| *UNK* | 28.9±1.5 | 27.5±0.8 | 28.1±1.2 | 27.5±0.4 | 29.1±2.1 | 29.1±2.1 |
| *18S rRNA* | 22.7±6.0 | 25.5±3.8 | 22.8±4.7 | 22.6±6.2 | 25.4±1.3 | 22.7±6.3 |
| *25S rRNA* | 8.7±0.5 | 8.8±0.3 | 9.7±0.9 | 8.7±0.1 | 11.2±2.8 | 10.6±2.5 |

Data represent average Ct values±SD for each condition from three pearl millet genotypes in three biological replicates. ^a^ Mannitol treatment.
